# Supplementary material for: Information transfer in QT-RR dynamics: Application to QT-correction
Source: Sci Rep. 2018 Oct 9;8:14992. doi: 10.1038/s41598-018-33359-1 (PMC6178346; doi:10.1038/s41598-018-33359-1)
Supplement: Supplementary file 1 — Supplementary Information [file 41598_2018_33359_MOESM1_ESM.pdf]

Information transfer in QT-RR dynamics: Application to QT-correction. Supplementary Information

Ilya Potapov[1,*], Joonas Latukka[1], Jiyeong Kim[1], Perttu Luukko[1,2], Katriina Aalto-Setälä[3,4], and Esa Räsänen[1]

[1]Laboratory of Physics, Tampere University of Technology, P.O. Box 692, FI-33101, Finland

[2]FirstBeat Technologies Ltd, Yliopistonkatu 28 a, Jyväskylä, 40100, Finland

[3]Heart Group, Faculty of Medicine and Life Sciences, University of Tampere, P.O. Box 100, FI-33014, Finland

[4]Heart Hospital, Tampere University Hospital, Finland

[*]ilya.potapov@tut.fi

Contents

S1 Data **2**

S1.1 Data acquisition and preprocessing . . . 2

S1.2 Recording length statistics . . . 2

S1.3 Consecutive beat segments of ECG . . . 3

S2 Information transfer asymmetry: variance study **5**

S3 Synthetic data **5**

S3.1 White noise . . . 5

S3.2 Pink noise . . . 7

S3.3 Auto-regressive filter . . . 9

S3.4 Auto-regressive filter with QT-correction . . . 11

S4 Information transfer and QT-correction **13**

S4.1 Bazett correction formula . . . 13

S4.2 QT-correction with average RR history . . . 14

S4.3 QT correction with exponentially weighted average of RR history . . . 20

S5 Information transfer and gender **29**

S6 Information transfer in presence of a third process **30**

S7 List of Figures **32**

S1 Data

S1.1 Data acquisition and preprocessing

Each ECG recording has been annotated as to determine locations of the Q, T, and R-waves using WFDB Toolbox (Matlab/Octave interface, `https://physionet.org/physiotools/matlab/wfdb-app-matlab/`). The Q-wave was determined as the beginning of the QRS complex. The waves belonging to the same heartbeat were extracted, from these the QT and RR intervals were calculated.

Segments of ECG containing more than 5 consecutive heartbeats were taken. These segments were joined to form a single time series for each subject. Importantly, this approach might affect the conclusions regarding the history length. See Sec. S1.3 where the segments with only consecutive heartbeats were analyzed.

During preprocessing the heartbeats with the RR interval less than 300 ms and larger than 1500 ms were discarded. Similarly, the heart beats with the QT interval less than 50 ms and larger than 600 ms were discarded. Additionally, the heartbeat was discarded if the change between consecutive RR/QT values was larger than 200/35 ms. If either RR or QT did not pass the cleaning criteria the whole heartbeat was discarded.

S1.2 Recording length statistics

After the preprocessing the 18 recordings had: minimum number of heart beats = 69, max = 26384, mean = 6512.2, median = 4538. The clear outliers with the minimal number of samples 69 and 380 were shown not to affect the qualitative results of the relation between transfer entropy (TE) and history length (see Fig. S1, cf. Fig. 3 of the main text). The group without the two outliers had: minimum number of beats = 1390, mean = 7298.1, median = 4697.

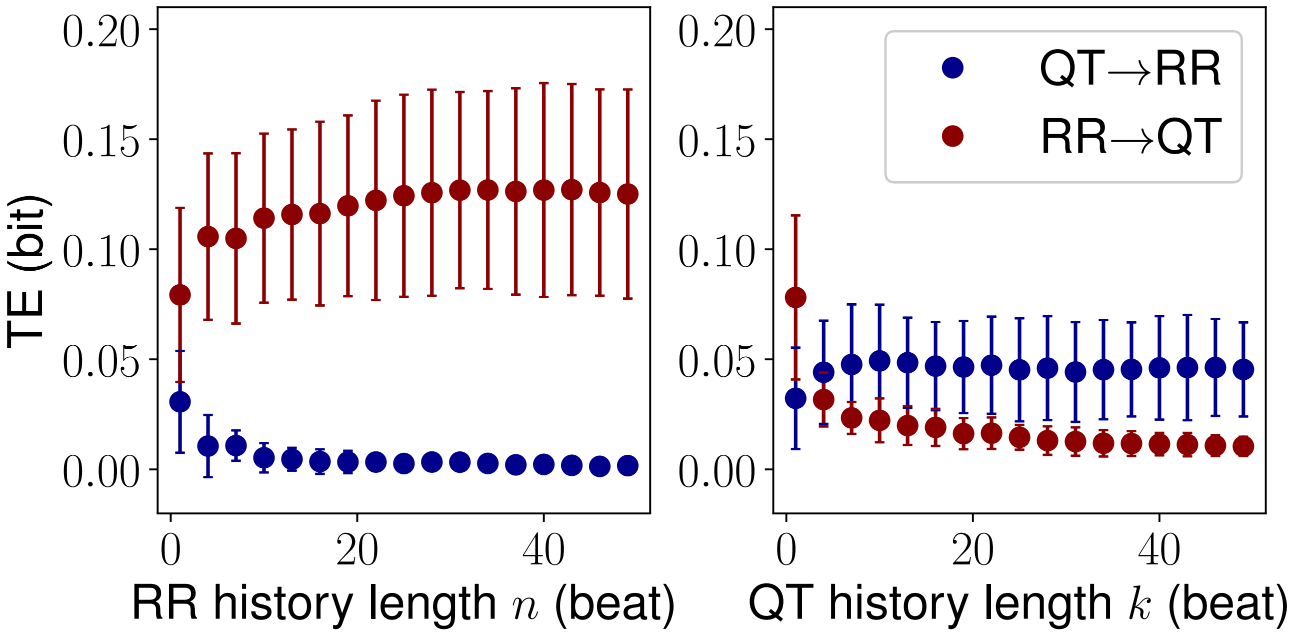

Figure S1: History length effect on information transfers. Dots (•) represent mean values, whereas the error bars standard deviations. Two sample size outliers with the minimal number of time samples 69 and 380 were removed, cf. Fig. 3 of the main text. Number of subjects is 16.

S1.3 Consecutive beat segments of ECG

To avoid concatenation of many consecutive heartbeat ECG segments from different time moments when considering the RR/QT history, we have extracted consecutive heartbeat segments from all subjects as separate time series. Each segment is guaranteed to be at least L_{min} heartbeats long. The results for different L_{min} are presented in Fig. S2.

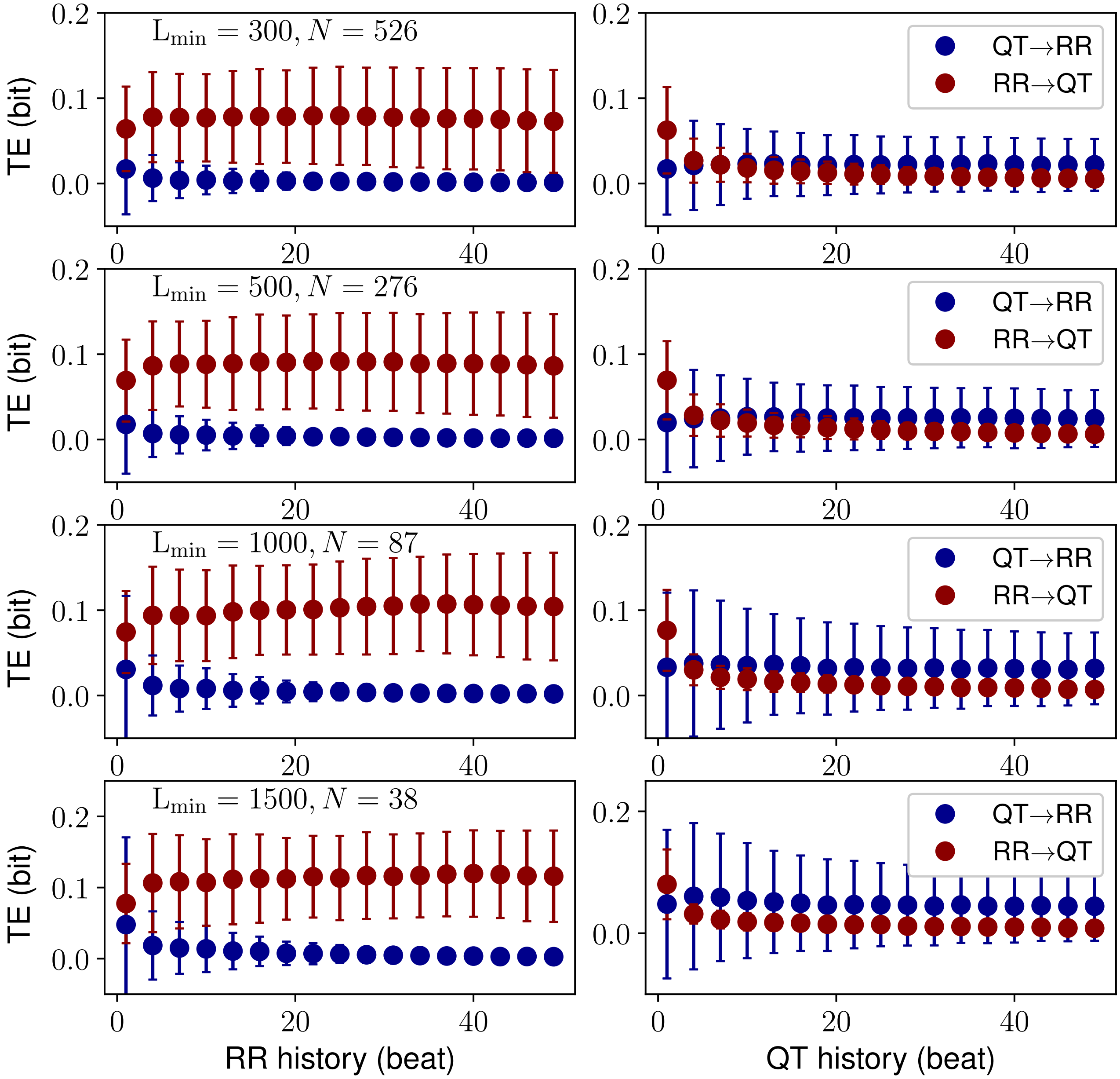

Figure S2: History length effect when consecutive ECG segments were analyzed as independent time series. Each row shows $TE_{RR \to QT}$ and $TE_{QT \to RR}$ over changing RR (n) and QT (k) history lengths for a fixed parameter L_{min}, a minimal number of heartbeats in each coupled time series. The number of time series ("group") is N.

One can still observe the asymmetry between $TE_{RR \to QT}$ and $TE_{QT \to RR}$ over varying n (cf. Figure 3 of the main text). The asymmetry is not obvious when the QT history k is varied (Fig. S2). Also, note the smaller group size when L_{min} increases.

S2 Information transfer asymmetry: variance study

We run transfer entropy (TE) calculations for 100 times, each time taking the average (Fig. 2 of the main text) and standard deviation of TE from 18 time series of the subject group. The resulting standard deviation distributions for RR→QT and QT→RR information transfers are shown in Fig. S3.

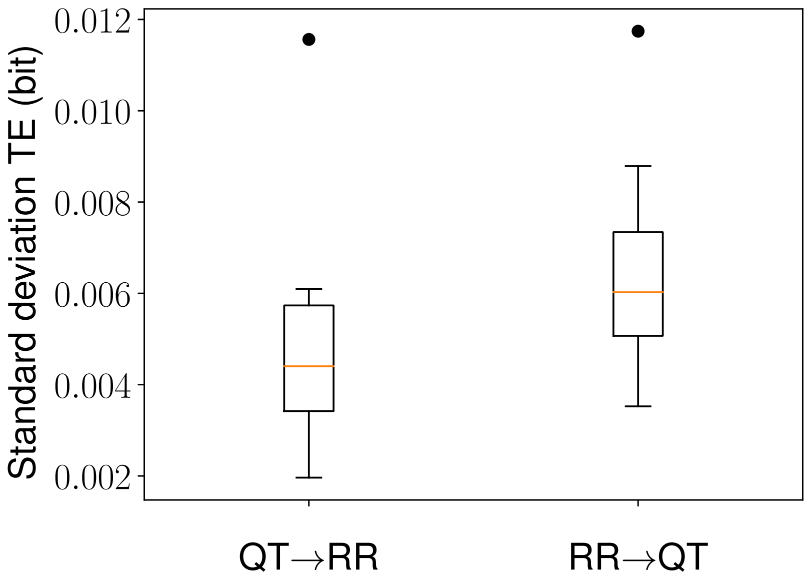

Figure S3: Distribution of the group TE standard deviations after 100 runs (unpaired two-sided t-test $P = 0.029$). Each run the standard deviation of TE values over the subject group is taken to form these distributions. History length $n = k = 1$. See the complementary Fig. 2 of the main text.

S3 Synthetic data

We use surrogate data to test the transfer entropy method. In this section we generate 20 synthetic RR and QT sequences ("subject group") each of 4000 values ("heart beats"). Unless specified otherwise the technical details of the computation are the same as in Methods of the main text.

S3.1 White noise

To mimic QT-RR relationship we generate RR sequence to follow the normal distribution with mean equal 800 ms and standard deviation 50 ms (that is, $\mathcal{N}(800, 50)$). Then QT $= 300/(RR/1000)^{\frac{1}{3}}$. We vary the history length of RR (n) and QT (k) sequences independently and for each history value calculate TE (see Fig. S4).

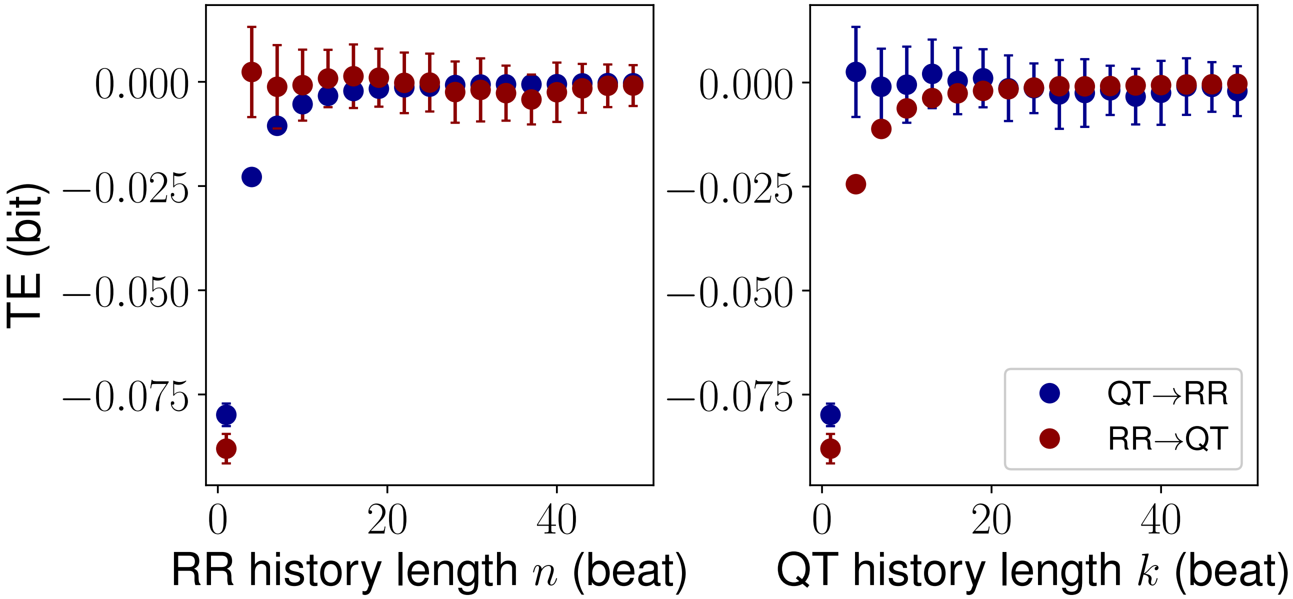

Figure S4: Synthetic data (RR $= \mathcal{N}(800 \text{ ms}, 50 \text{ ms})$, QT $= 300/(RR/1000)^{\frac{1}{3}}$): History length effect on information transfers. $k = 1$ and $n = 1$ on the left and right panels, respectively. Dots (•) represent mean values, whereas the error bars standard deviations.

Note the substantially negative values of TE for history of one beat. This is due to the probability estimation statistical errors (Kraskov-Stögbauer-Grassberger, KSG, probability estimation algorithm no. 1), which can be avoided by increasing the number of nearest neighbors to 100 (Fig. S5).

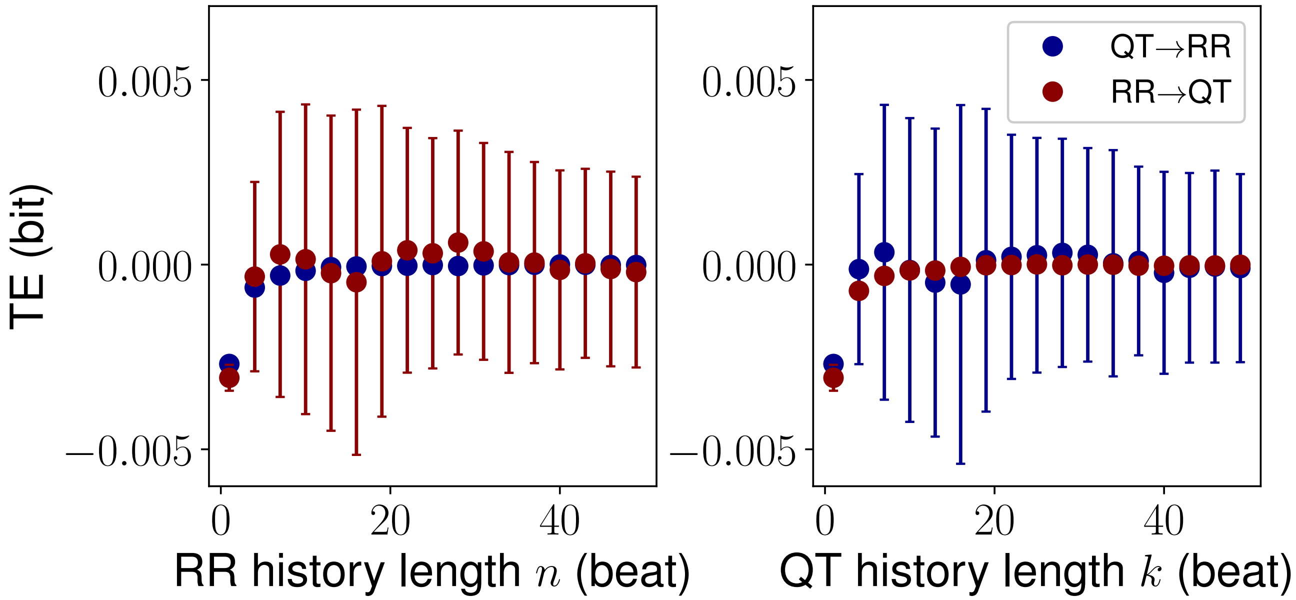

Figure S5: Same as in Fig. S4, but the number of nearest neighbors in the Kraskov-Stögbauer-Grassberger algorithm is 100.

Figure S5 reveals no inter-dependence between RR following normal random distribution and QT, corrected using the Fridericia formula and fixed QT mean value.

S3.2 Pink noise

We can generate RR sequence to follow the pink noise (time series with long-range correlations, mean equal 800 ms, and standard deviation 50 ms, that is RR $= \mathcal{P}(800\text{ ms}, 50\text{ ms})$), which is shown in many studies to be more realistic (see, e.g., [Peng et al., *Chaos*, 5:82–87, 1995]). We again assume QT $= 300/(RR/1000)^{\frac{1}{3}}$. The history effects on the transfers are shown in Fig. S6.

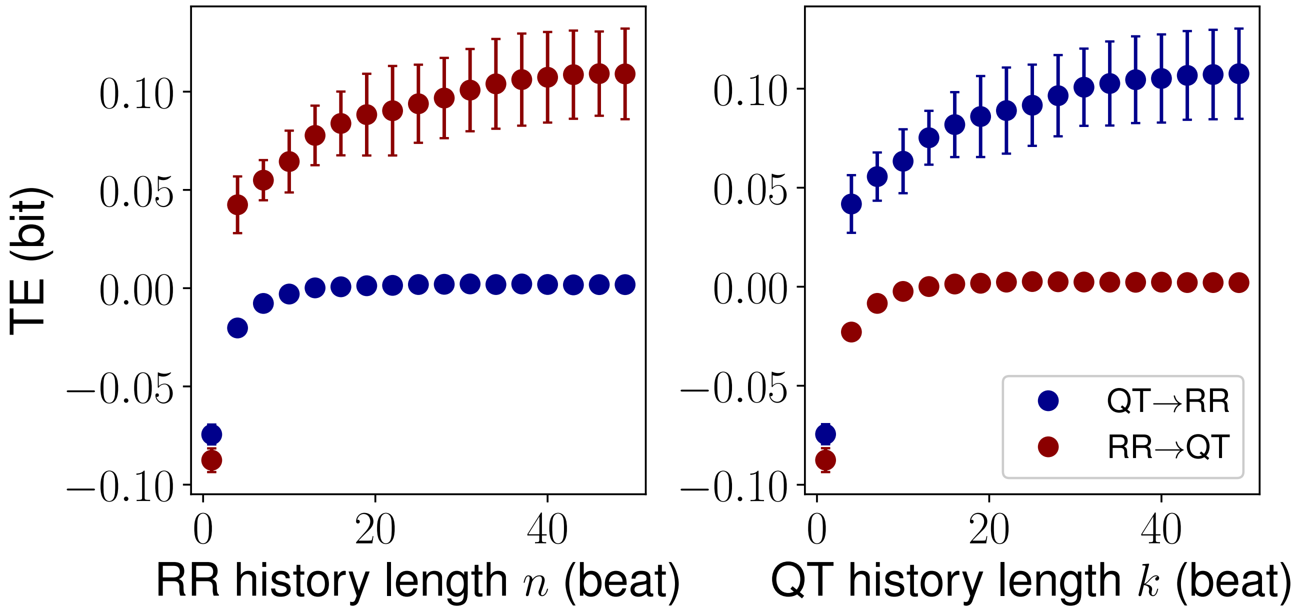

Figure S6: Synthetic data (RR $= \mathcal{P}(800\text{ ms}, 50\text{ ms})$, QT $= 300/(RR/1000)^{\frac{1}{3}}$): History length effect on information transfers. $k = 1$ and $n = 1$ on the left and right panels, respectively. Dots (•) represent mean values, whereas the error bars standard deviations.

The result of increasing the number of nearest neighbors to 100 in KSG algorithm is shown in Fig. S7.

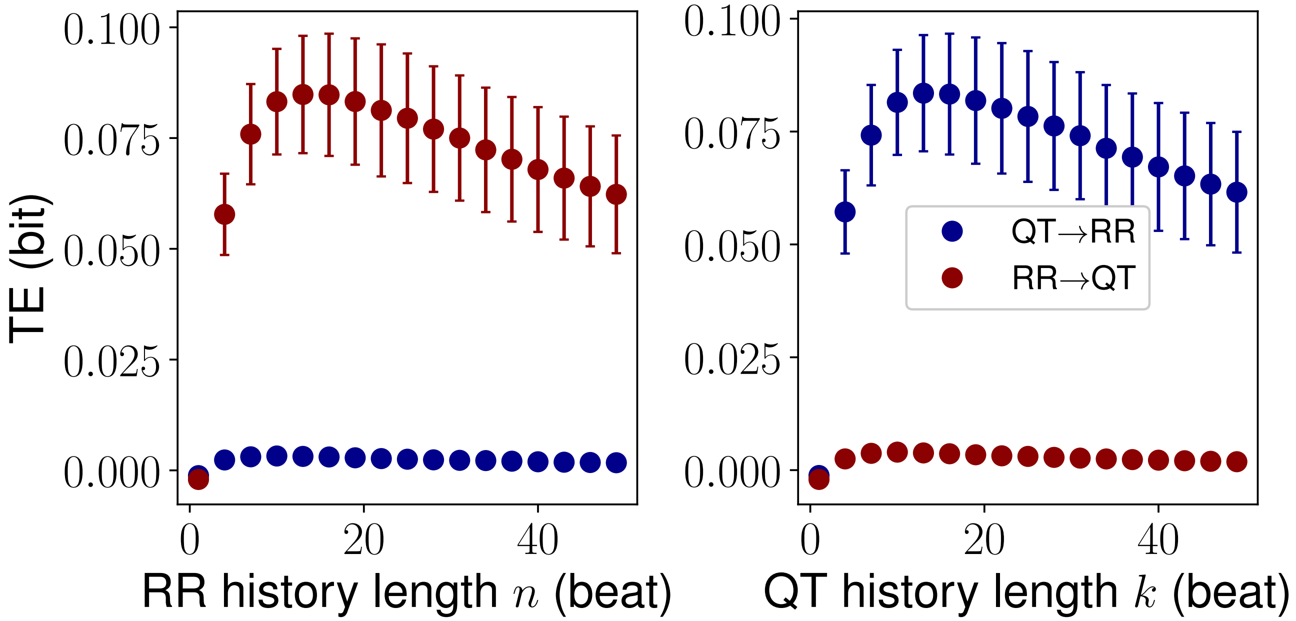

Figure S7: Same as in Fig. S6 but with 100 nearest neighbors of the KSG algorithm.

One can see that the pink-noise generated RR and QT sequences generate information flows more closely following

those of the real QT and RR sequences of healthy individuals (cf. Fig. 3 of the main text). However, there is no asymmetry in the information flows and $TE_{RR \to QT}$ under varying n is (almost) identical to $TE_{QT \to RR}$ under varying k (Fig. S7).

S3.3 Auto-regressive filter

We assume $RR = \mathcal{N}(800 \text{ ms}, 50 \text{ ms})$ and

$$QT_i = \sum_{m=1}^{M} a_m RR_{i-m} + \epsilon_i \,,$$

where ϵ_i is an error term. The parameters a_m are estimated using Yule-Walker equation for different M. KSG algorithm for the TE calculation uses 100 nearest neighbors. (Note: the QT values are not realistic in this approach and follow closely the RR interval values.)

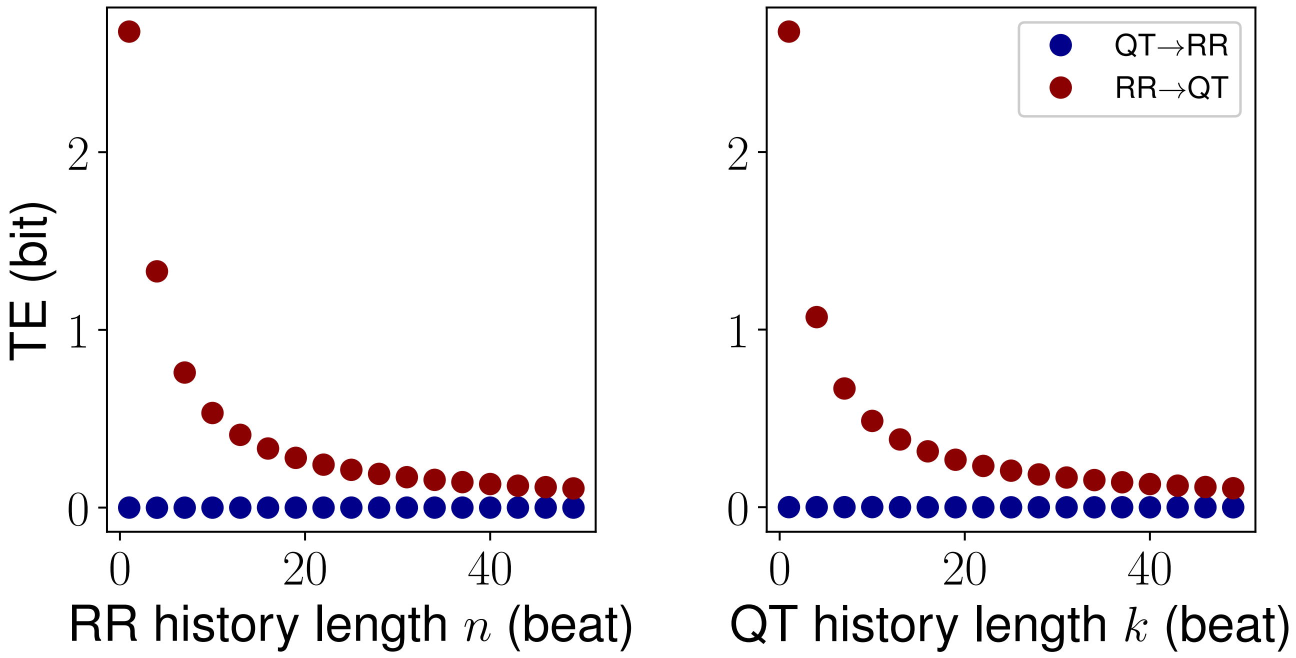

Figure S8: Synthetic data ($RR = \mathcal{N}(800 \text{ ms}, 50 \text{ ms})$, $QT_i = \sum_{m=1}^{M} a_m RR_{i-m} + \epsilon_i$, $M = 1$): History length effect on information transfers. $k = 1$ and $n = 1$ on the left and right panels, respectively. Dots (•) represent mean values, whereas the error bars standard deviations.

RR→QT transfer is decaying over both n and k history length for $M = 1$ (Fig. S8). This shows the importance of the recent history values in determining QT. Noteworthy, QT→RR is always zero in this approach indicating that there is no information flow from QT to RR as is the case here.

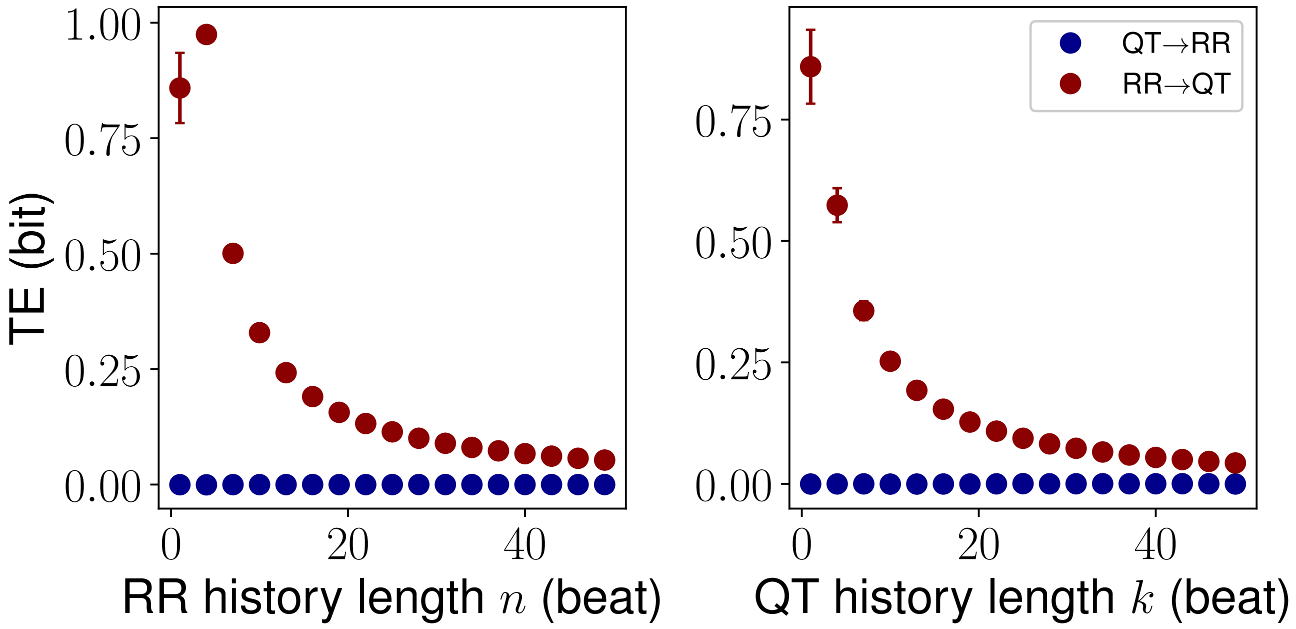

Figure S9: Same as in Fig. S8, but $M = 2$.

Similar trends of $\mathrm{TE}_{\mathrm{RR}\to\mathrm{QT}}$ one can observe for $M = 2$ (Fig. S9). However, the absolute value of the RR→QT transfer is lower than for $M = 1$. As for $M = 1$ $\mathrm{TE}_{\mathrm{QT}\to\mathrm{RR}}$ is effectively zero.

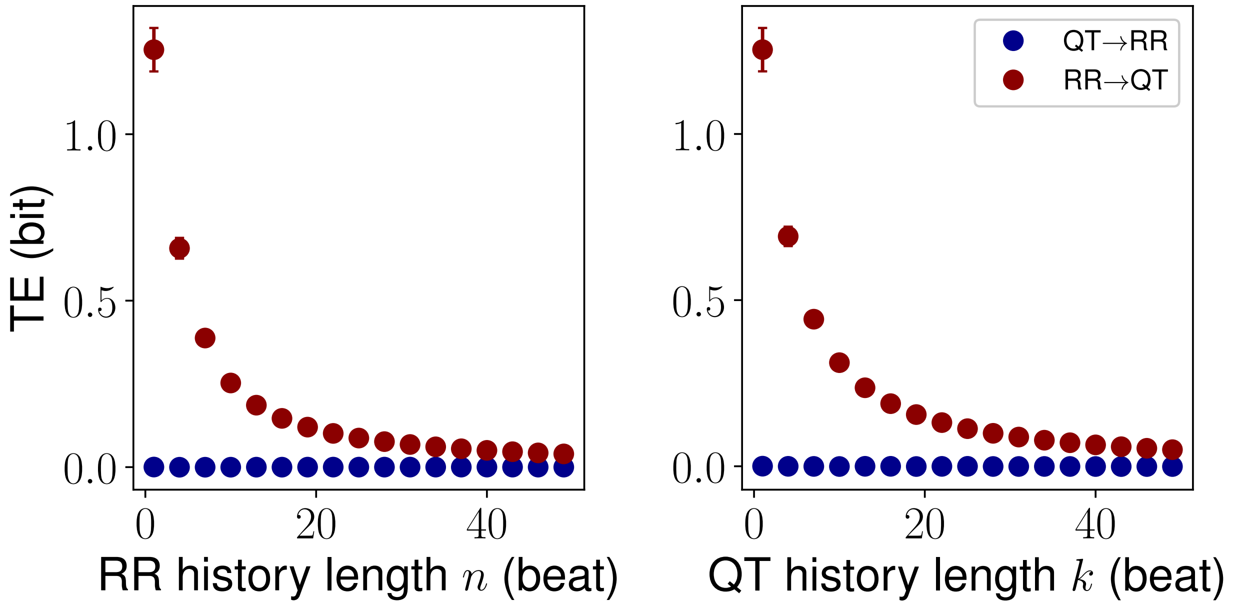

Figure S10: Same as in Fig. S8, but $M = 5$.

For $M = 5$ the transfer absolute values are comparable with those for $M = 2$ (Fig. S10). This might suggest, in the case of $\mathrm{TE}_{\mathrm{RR}\to\mathrm{QT}}$, that the very recent history contributes the most to determining QT values.

S3.4 Auto-regressive filter with QT-correction

We assume $\mathrm{RR} = \mathcal{N}(800\ \mathrm{ms}, 50\ \mathrm{ms})$ and

$$\mathrm{QT_i} = \sum_{m=1}^{M} a_m \mathrm{RR}_{i-m} + \epsilon_i\,.$$

where ϵ_i is an error term. Then, QT series is modified according to the Bazett QT-correction formula:

$$\mathrm{QTcB_i} = \frac{\mathrm{QT_i}}{\sqrt{\mathrm{RR_i}/1000}}$$

The information transfer is calculated between QTcB and RR series.

The parameters a_m are estimated using Yule-Walker equation for different M. KSG algorithm for the TE calculation uses 100 nearest neighbors. (Note: the QT values are not realistic in this approach and follow closely the RR interval values.)

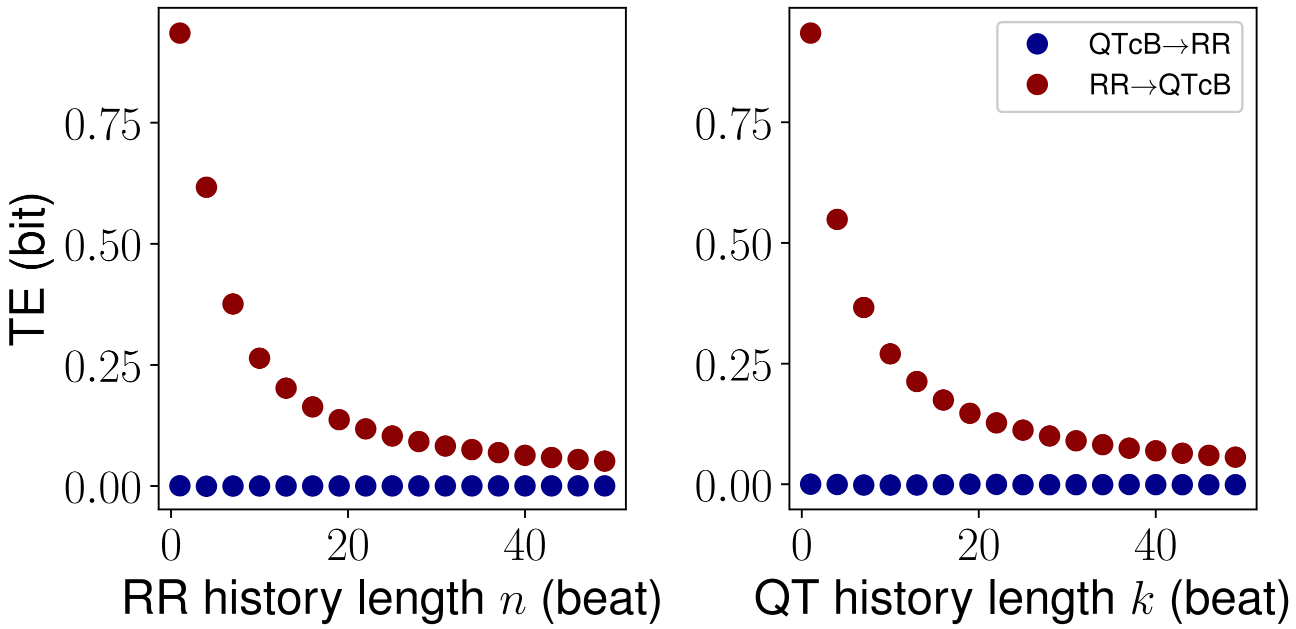

Figure S11: Synthetic data ($\mathrm{RR} = \mathcal{N}(800\ \mathrm{ms}, 50\ \mathrm{ms})$, $\mathrm{QT_i} = \frac{\sum_{m=1}^{M} a_m \mathrm{RR}_{i-m} + \epsilon_i}{\sqrt{\mathrm{RR_i}/1000}}$, $M = 1$): History length effect on information transfers. $k = 1$ and $n = 1$ on the left and right panels, respectively. Dots (•) represent mean values, whereas the error bars standard deviations.

$\mathrm{TE_{RR\to QT}}$ is decaying over both n and k history length for $M = 1$ (Fig. S11). Noteworthy, QT→RR is always zero in this approach indicating that there is no information flow from QT to RR. Note that the absolute value of $\mathrm{TE_{RR\to QT}}$ is lower than in the corresponding case without QT-correction (Fig. S8), this might indicate the reduction in the information flow due to the QT-correction.

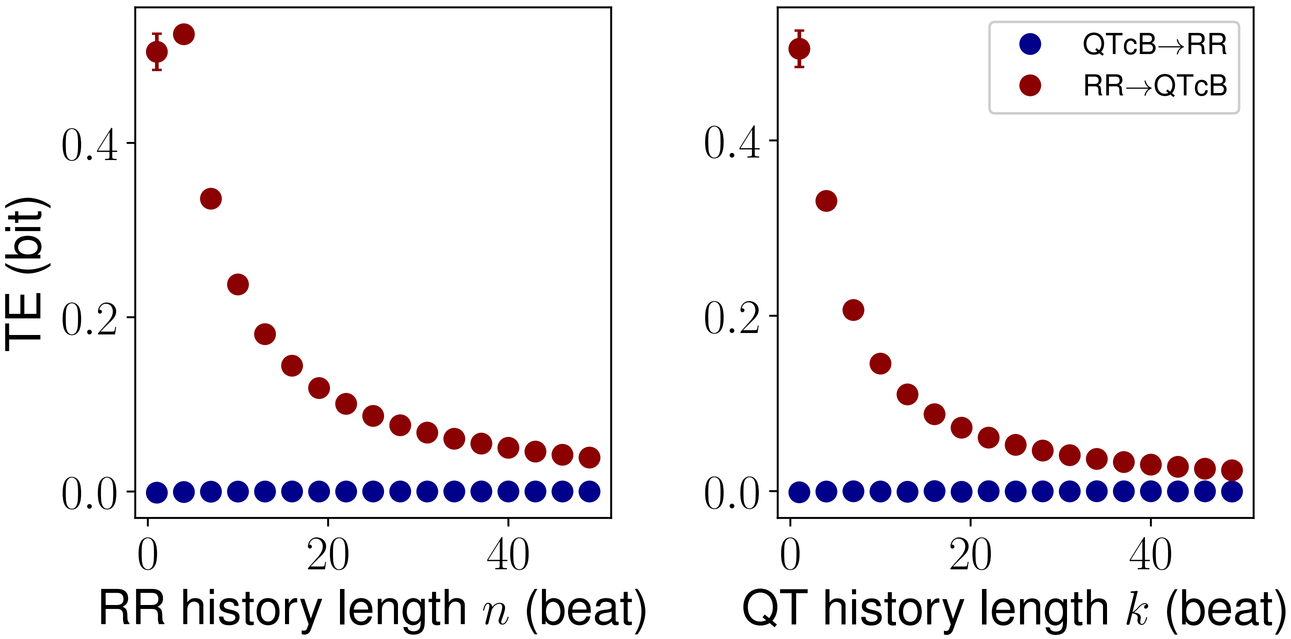

Figure S12: Same as in Fig. S11, but $M = 2$.

Similar trends of both transfers are observed for $M = 2$ (Fig. S12). However, the absolute value of the RR→QT transfer is lower than for $M = 1$ (Fig. S11). Similarly $TE_{QT \to RR}$ is effectively zero as for $M = 1$.

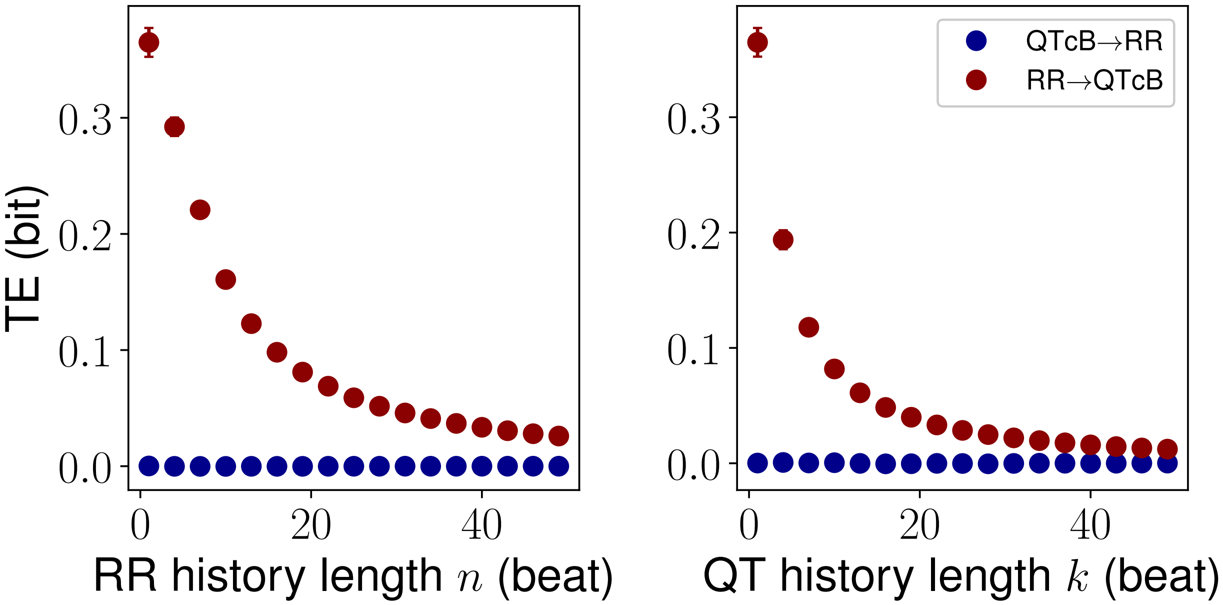

Figure S13: Same as in Fig. S11, but $M = 5$.

For $M = 5$ the transfer absolute values are lower than those for $M = 2$ (Fig. S13). This might suggest, the contribution of the QT-correction procedure in reducing the value of $TE_{RR \to QT}$, given that the corresponding non-

corrected values (Fig. S10) were comparable with the $M = 2$ case (Fig. S9).

S4 Information transfer and QT-correction

S4.1 Bazett correction formula

The Bazett correction formula is $\mathrm{QTc} = \dfrac{\mathrm{QT}}{\sqrt{\mathrm{RR}/1000}}$ (QT and RR in milliseconds). It is important to note that QTc depends on two variables: the measured QT and RR, hence it is a surface over QT-RR plane (the same applied to the Fridericia correction formula). The QT-RR point cloud for a single subject is shown in Fig. S14.

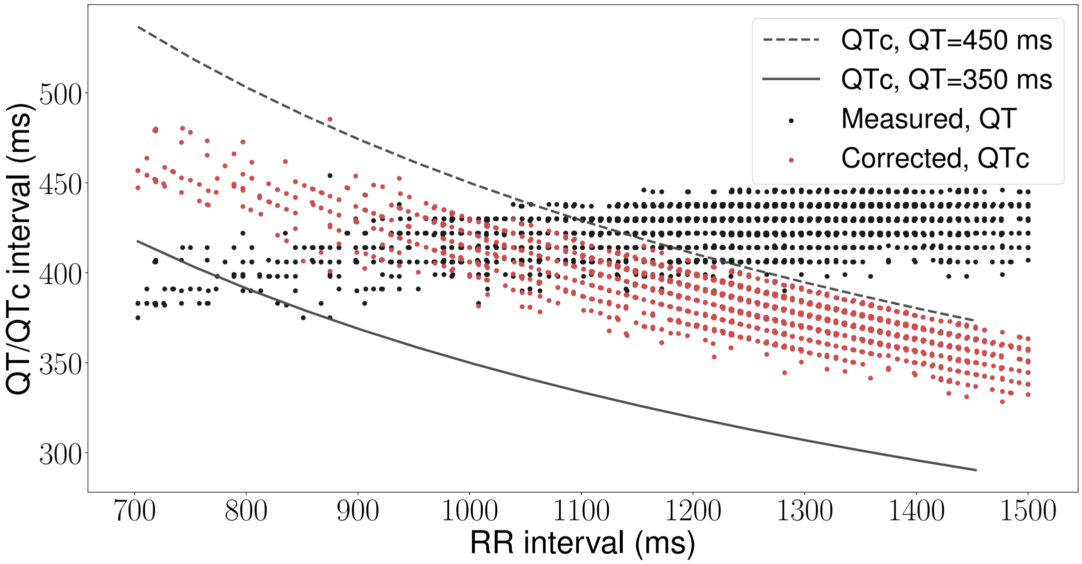

Figure S14: A sample measured QT-RR point cloud (black) and its QT-corrected counterpart (red) with two lines showing how the Bazett formula behaves for a *fixed* QT value.

The effect of the QT-correction on the original data according to the Bazett formula is shown in Fig. S15 (cf. the complementary Fig. 7 of the main article, where we used the QT-correction according to the Fridericia formula).

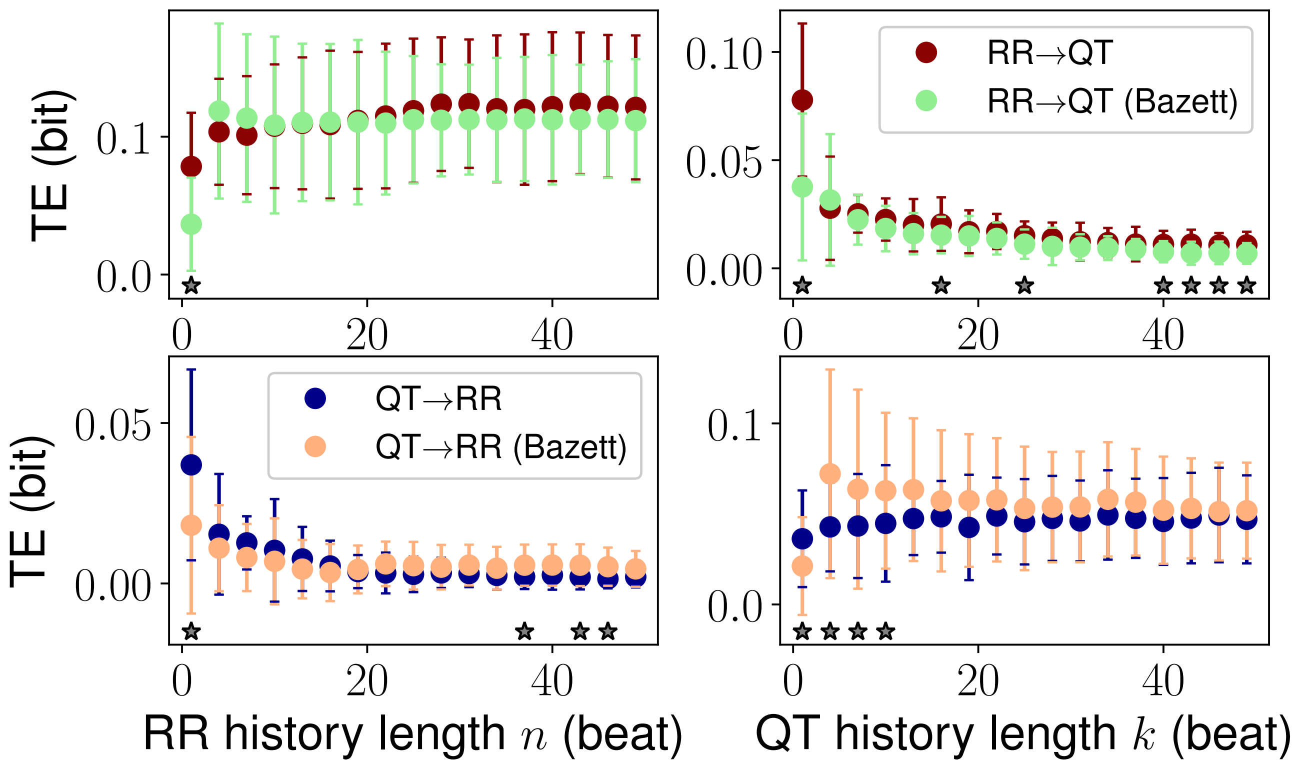

Figure S15: The effect of the QT correction (Bazett formula) on the information flows. The stars show significant ($P \leq 0.05$, paired t-test) differences between TE distributions of the original and corrected signals. Data in the format mean ± standard deviation (cf. Fig. 7 of the main text).

S4.2 QT-correction with average RR history

We use the average of the preceding RR interval values in the QT-correction formulas (all RR and QT values are in milliseconds in this section). Namely, for the Bazett QT-correction formula:

$$\mathrm{QTc_i} = \frac{\mathrm{QT_i}}{\sqrt{\mathrm{RR_{avg}}/1000}}$$

where $\mathrm{RR_{avg}}$ is calculated as:

$$\mathrm{RR_{avg}} = \frac{\sum_{m=1}^{M} \mathrm{RR_{i-m}}}{M}$$

The results are presented below for different averaging window sizes M (5, 10, 20, and 40 heartbeats).

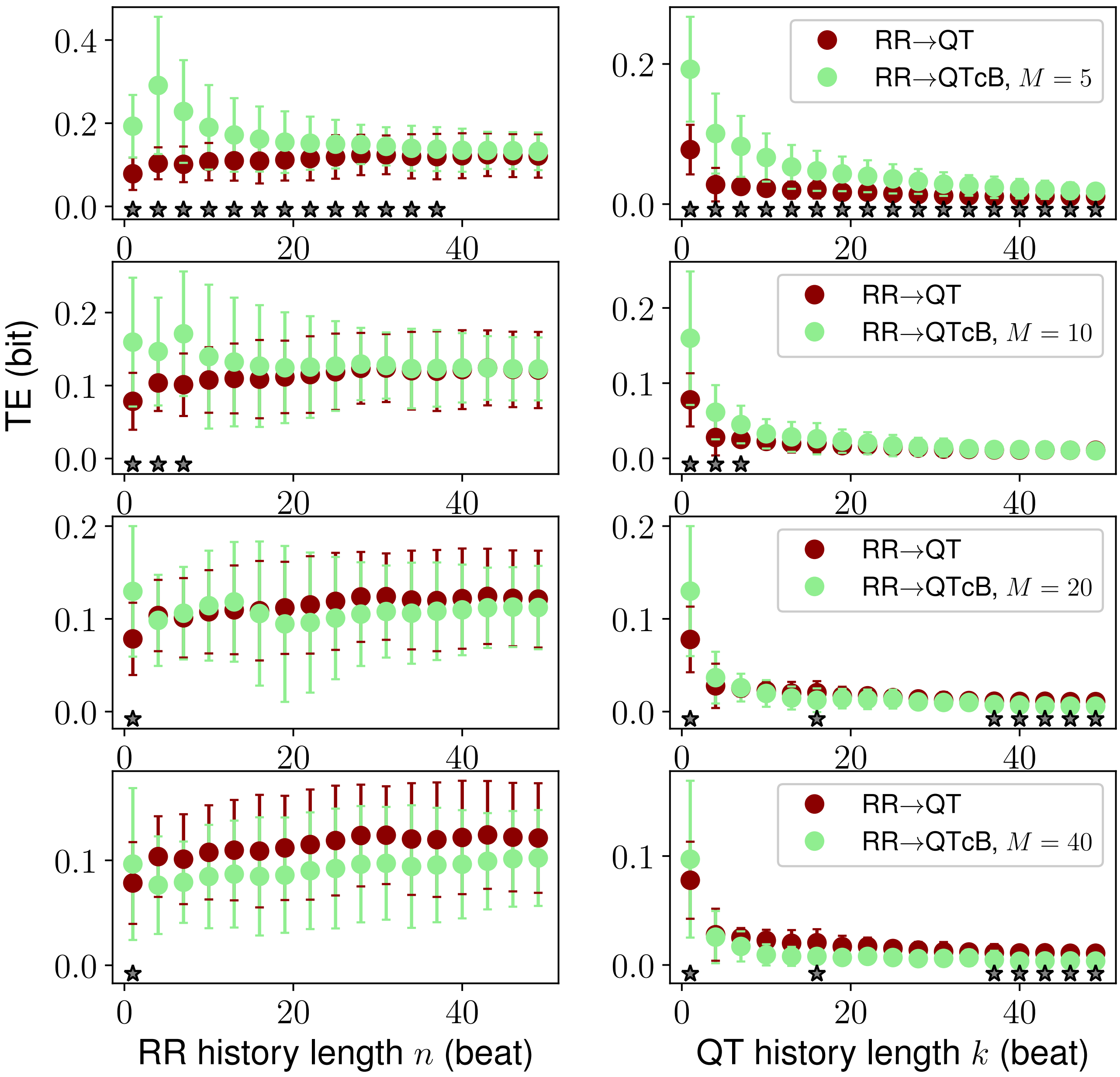

Figure S16: RR→QTcB (QT-correction with the Bazett formula) information transfers using the RR history averaging model. The stars show significant ($P \leq 0.05$, paired t-test) differences between TE distributions of the original and corrected signals. Data in the format mean ± standard deviation (cf. Fig. 7 of the main text).

$\mathrm{TE}_{\mathrm{RR}\to\mathrm{QTcB}}$ is affected by different averaging window sizes M. Namely, the curve of $\mathrm{TE}_{\mathrm{RR}\to\mathrm{QTcB}}$ over n and k goes down toward zero indicating reduction in the QT dependence on RR, which is sought in the QT-correction procedure. Although reduction is visible, it is not enough to fully drop the transfer to zero (Fig. S16).

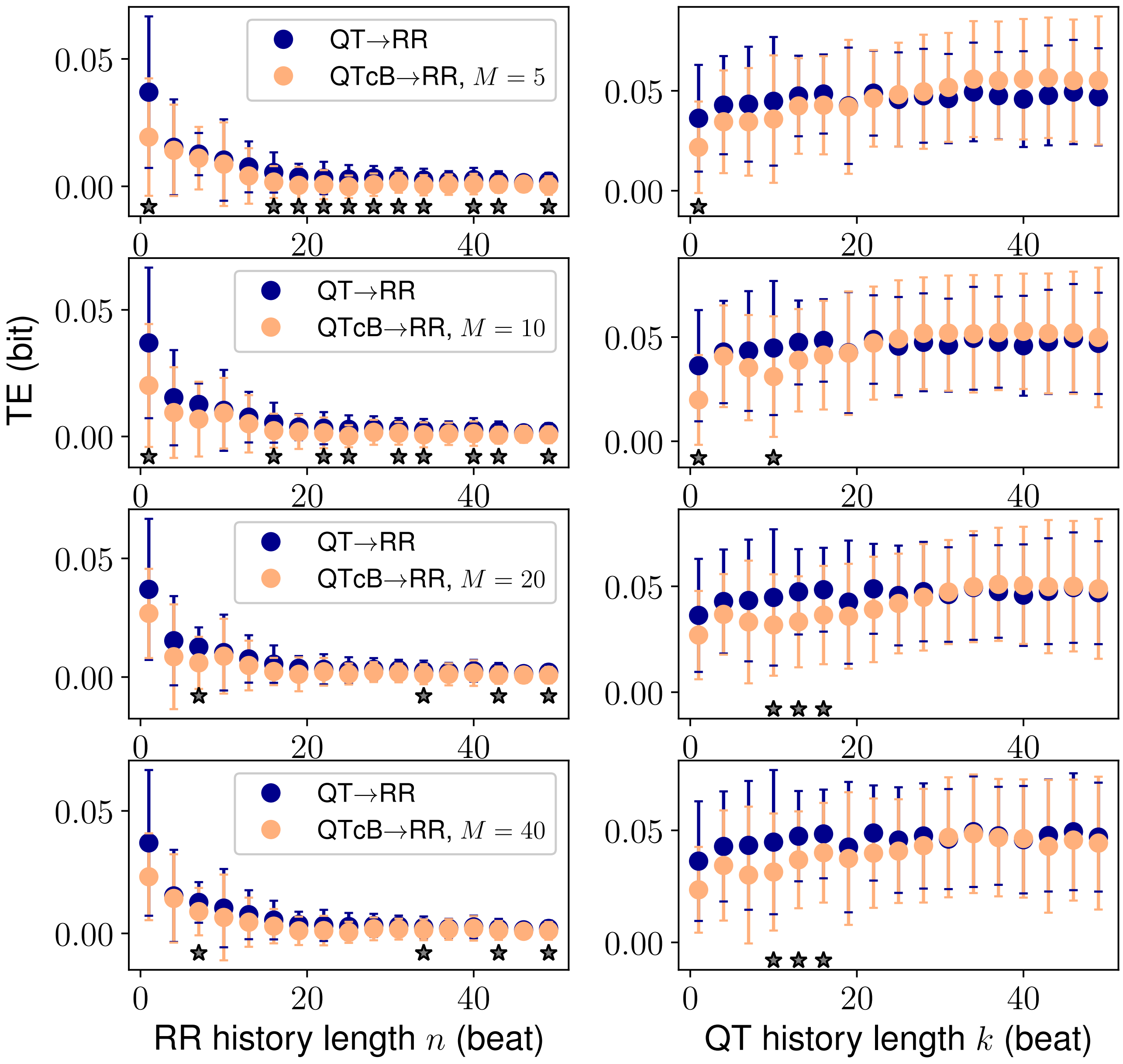

Figure S17: QTcB→RR information transfers using the RR history averaging model. The stars show significant ($P \leq 0.05$, paired t-test) differences between TE distributions of the original and corrected signals. Data in the format mean ± standard deviation (cf. Fig. 7 of the main text).

$\mathrm{TE}_{\mathrm{QTcB}\to\mathrm{RR}}$ reveals similar trends and in most cases is the same as the original $\mathrm{TE}_{\mathrm{QT}\to\mathrm{RR}}$ (Fig. S17), Similarly we calculate the information transfers using the Fridericia formula for the QT-correction:

$$\mathrm{QTc_i} = \frac{\mathrm{QT_i}}{(\mathrm{RR_{avg}}/1000)^{\frac{1}{3}}}$$

where $\mathrm{RR_{avg}}$ is calculated as:

$$\mathrm{RR_{avg}} = \frac{\sum_{m=1}^{M} \mathrm{RR_{i-m}}}{M}$$

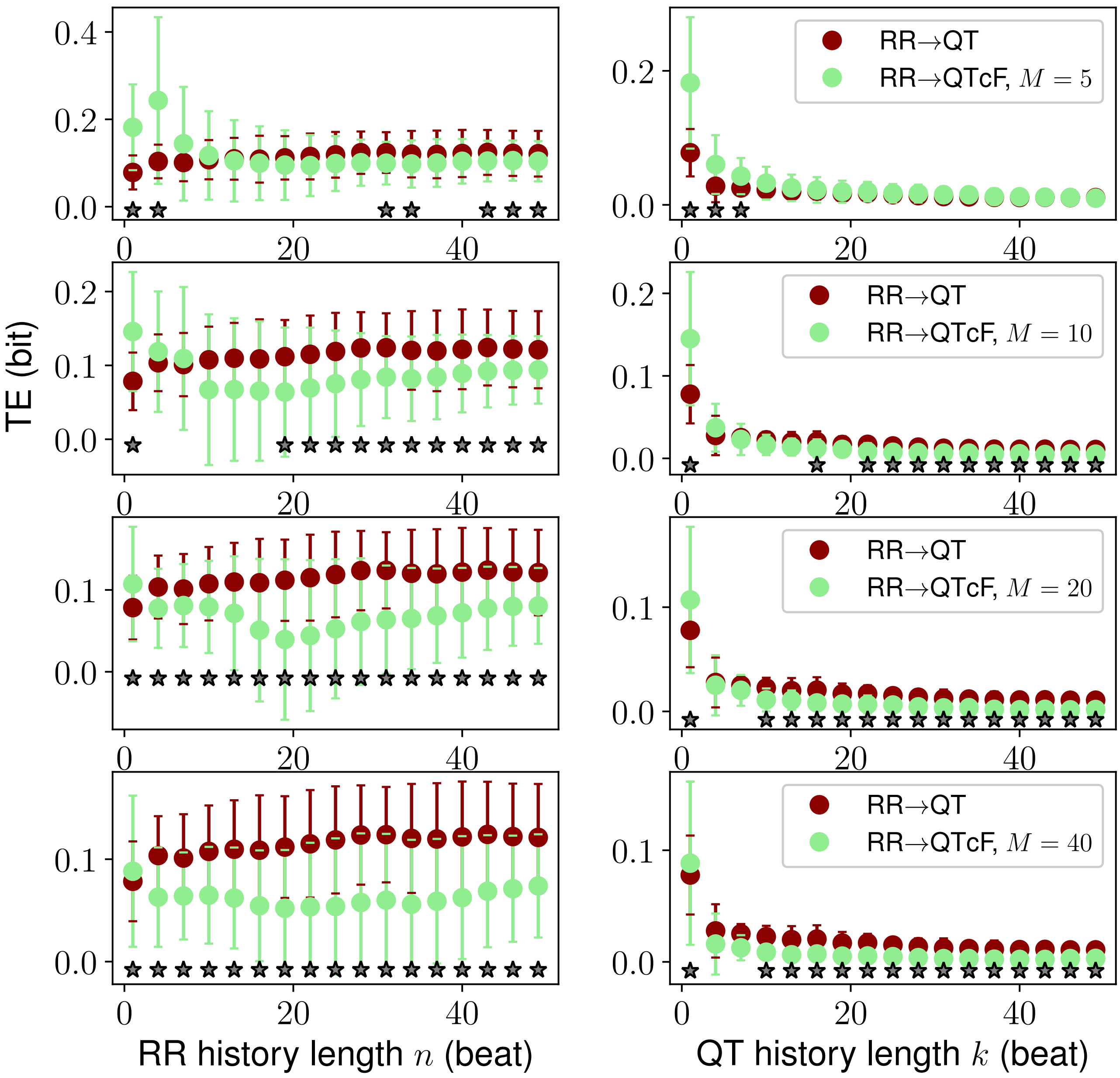

Figure S18: RR→QTcF (QT-correction with the Fridericia formula) information transfers using the RR history averaging model. The stars show significant ($P \leq 0.05$, paired t-test) differences between TE distributions of the original and corrected signals. Data in the format mean ± standard deviation (cf. Fig. 7 of the main text).

For the Fridericia QT-correction $\mathrm{TE_{RR \to QTcF}}$ also shows a similar drop both over n and k as in the case of the Bazett correction (Fig. S16). Moreover, the reduction in TE is more pronounced, suggesting the Fridericia correction

has advantages in reducing the QT dependency on RR. Noteworthy, for $M = 20$ the RR$\rightarrow$QTcF transfer has a clear minimum around $n = 20$ heart beats, suggesting (independently from the main text findings) that the healthy coupled QT-RR dynamics has internal characteristic history length of around 20 heartbeats. Importantly, this value is not connected to the averaging window size $M = 20$ as for $M = 40$ the minimum is still visible, but less pronounced at $n = 20$ beats (Fig. S18).

It is important to note here, that the 20 heartbeat history length may not necessarily be connected to the physiological parameters of the heart as during the preprocessing of the data multiple consecutive beat segments were *joined* to form a single time series for an individual. Nevertheless, this characteristic length consistently appeared in several tests indicating that such a critical length might exist (see also Sec. S1.3).

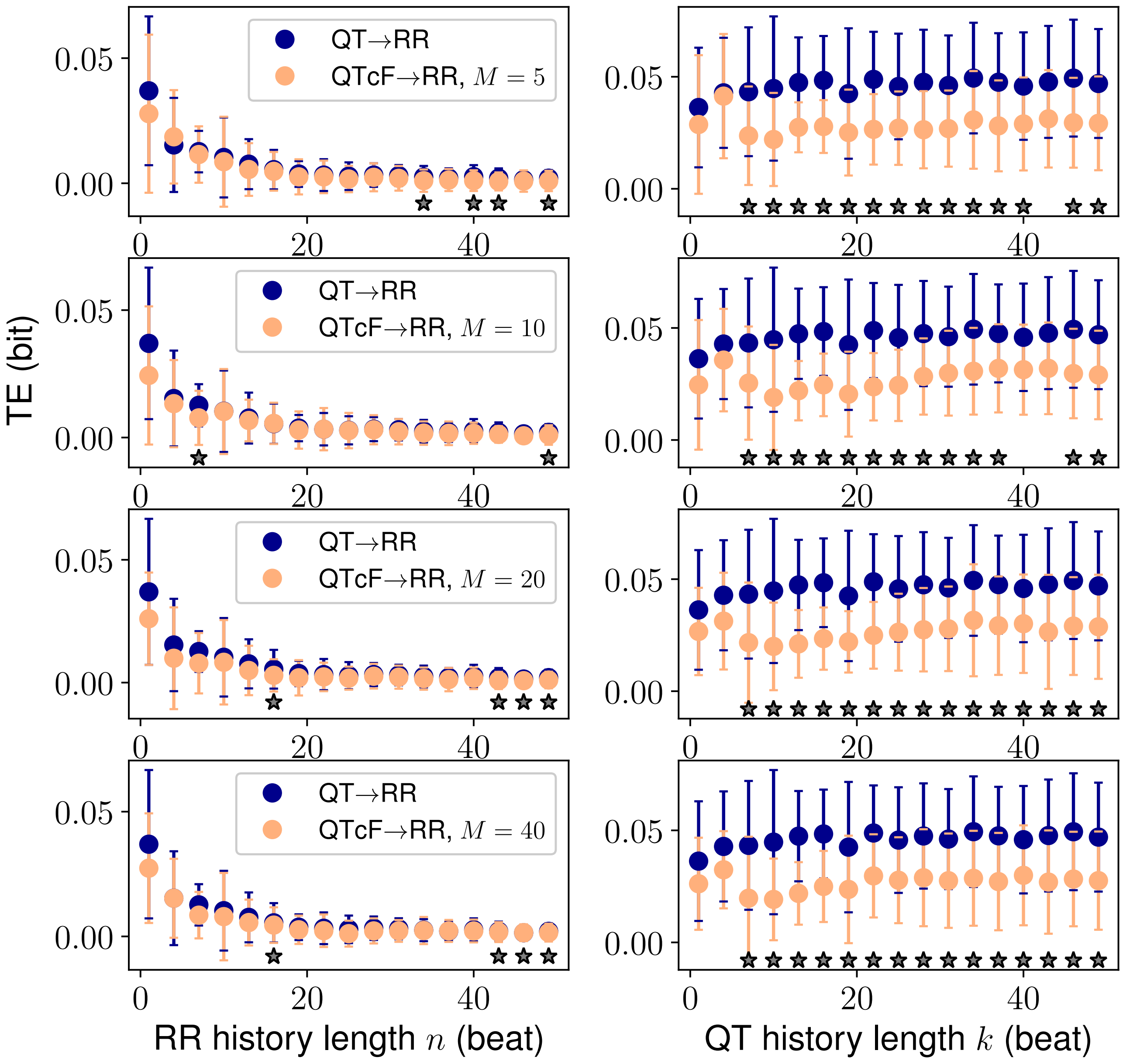

Figure S19: QTcF→RR information transfers using the RR history averaging model. The stars show significant ($P \leq 0.05$, paired t-test) differences between TE distributions of the original and corrected signals. Data in the format mean ± standard deviation (cf. Fig. 7 of the main text).

The information transfer value $\mathrm{TE}_{\mathrm{QTcF}\to\mathrm{RR}}$ does not undergo significant variation under varying M. Some systematic lower than original values of the transfer are observed over varying QT history k (Fig. S19), which was not observed in the case of Bazett QT-correction (Fig. S17).

S4.3 QT correction with exponentially weighted average of RR history

Next, we consider the class of exponentially weighted average models. These models take the exponentially weighted average of the RR history to contribute to the QT-correction. The model presented here is borrowed from [Riad et al. *PLoS One* 12, 1–14, 2017] (Eq. 1 therein).

The QT-correction formulas are:

$$\mathrm{QTcB_j} = \frac{\mathrm{QT_j}}{\sqrt{\mathrm{RR_{exp}}/1000}}\ ,\ \mathrm{QTcF_j} = \frac{\mathrm{QT_j}}{(\mathrm{RR_{exp}}/1000)^{\frac{1}{3}}}$$

where $\mathrm{RR_{exp}}$ is defined as follows:

$$\mathrm{RR_{exp}} = \mathrm{IR} \cdot \mathrm{RR_0} + (1 - \mathrm{IR})\frac{\sum_{i=-1}^{-M} \mathrm{RR_i} \cdot w_i}{\sum_{i=-1}^{-M} w_i}$$

where $\mathrm{RR_0}$ is the preceding beat RR interval, IR is the immediate response coefficient ranging from 0 (no contribution of the preceding beat) to 1 (full contribution of the preceding beat). The exponential weights $w_i = e^{-t_i/\tau}$, where t_i is the time elapsed from the end of the $\mathrm{RR_i}$ interval to the current j-th beat R-wave (the heartbeat of the QT being corrected), whereas τ is a time constant taking on values of 30, 60, and 120 s in this study (following [Riad et al., 2017]). The constant M is the number of history RR intervals defined so that the total history time t_i does not exceed 180 s (according to [Riad et al., 2017]).

A sample of the RR history and its corresponding weighted contribution are depicted in Fig. S20.

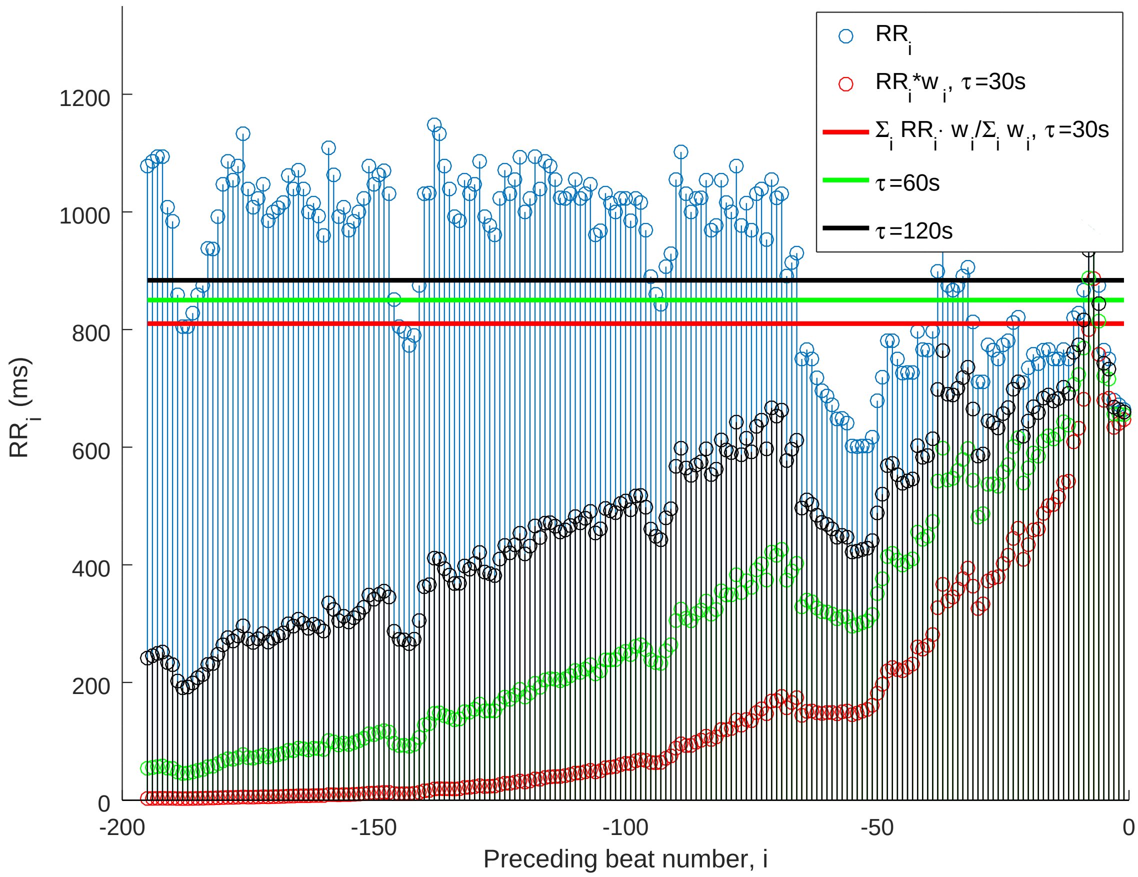

Figure S20: Sample representation of the weighted average contribution of the RR history. The true history RR intervals (blue) are taken with exponentially decaying weights corresponding to three different (red, green, and black) time constants τ of the model. The straight lines represent the average RR interval to be taken for the QT-correction.

Then, we calculate information transfers between RR $\rightarrow$ QT_c and QT_c $\rightarrow$ RR for IR $= 0.5$ and IR $= 0.183$ (following [Riad et al., 2017]).

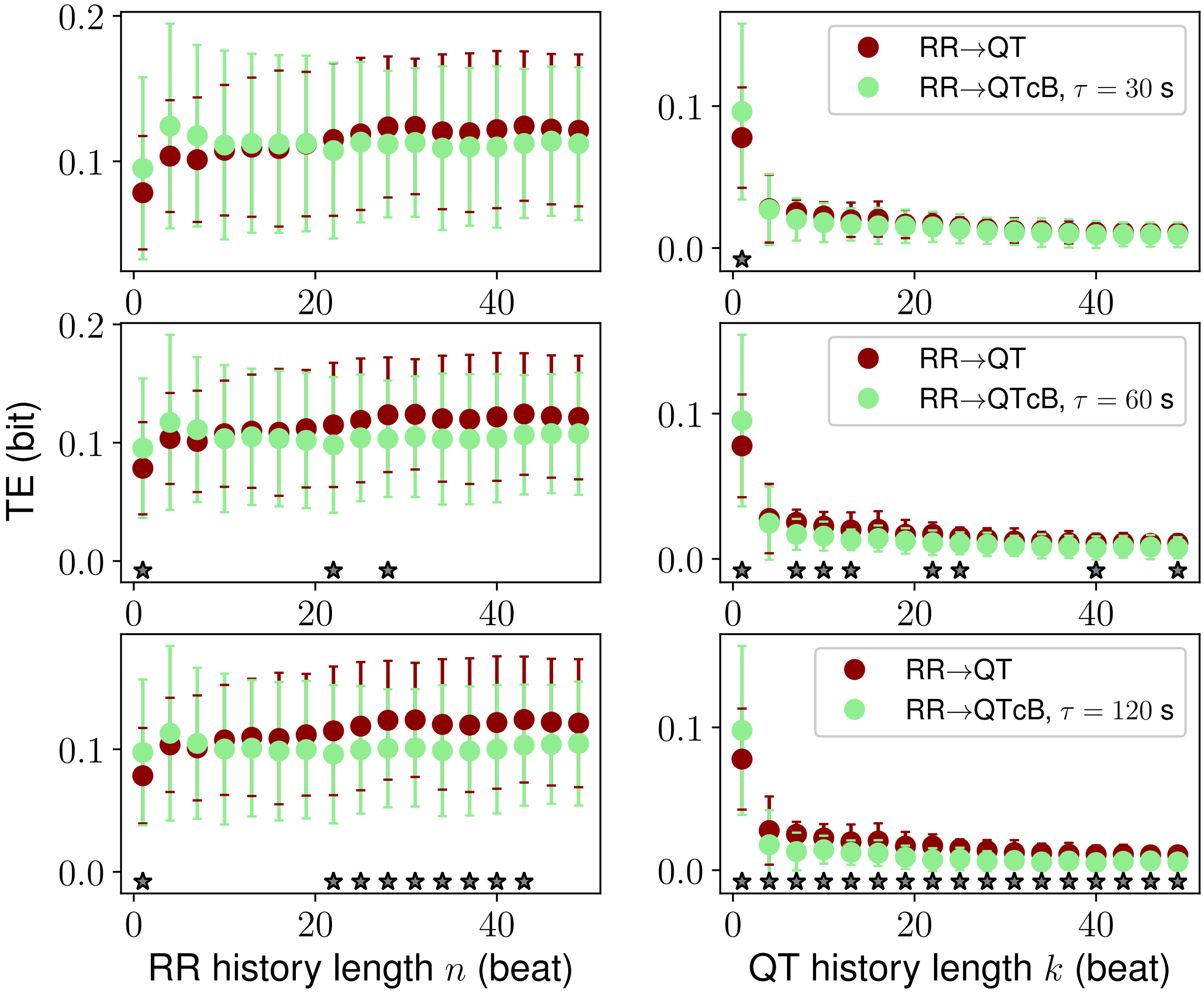

Figure S21: RR→QT and RR→QTcB (QT-corrected with the Bazett formula) transfers using the weighted average model (IR=0.5) for different time constants τ. The stars show significant ($P \leq 0.05$, paired t-test) differences between TE distributions of the original and corrected signals. Data in the format mean ± standard deviation

In the case of equal contribution from RR_0 and the history of RR, i.e. IR = 0.5, reduction of RR→QTcB is evident only for $n > 20$ and all k for $\tau = 120$ s. The larger τ facilitates more TE values to be reduced toward zero (Fig. S21).

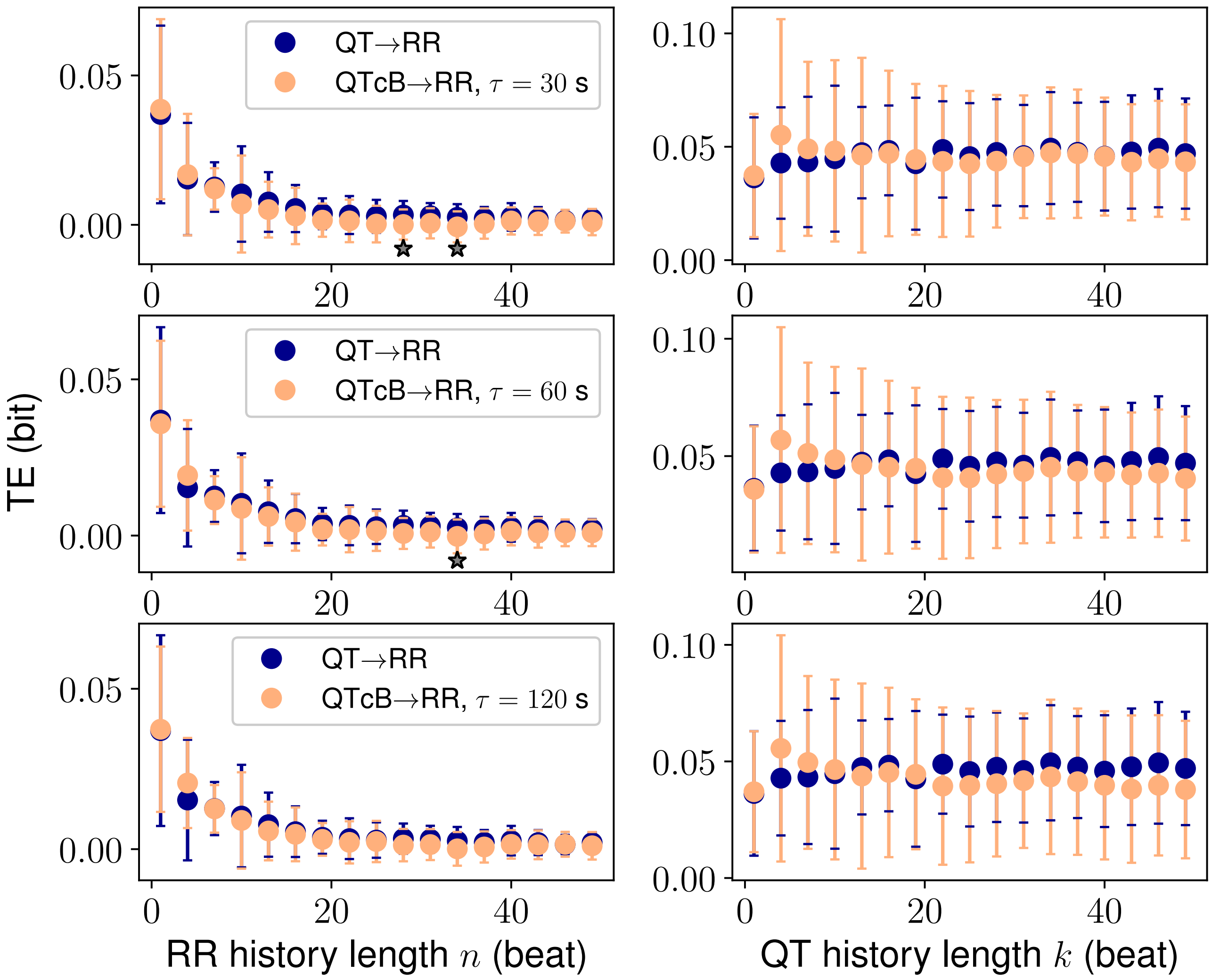

Figure S22: QT→RR and QTcB→RR (QT-corrected with the Bazett formula) transfers using the weighted average model (IR=0.5) for different time constants τ. The stars show significant ($P \leq 0.05$, paired t-test) differences between TE distributions of the original and corrected signals. Data in the format mean ± standard deviation

$\mathrm{TE}_{\mathrm{QTcB}\rightarrow\mathrm{RR}}$ is indistinguishable from the original transfer for almost all n and k, given IR = 0.5 (Fig. S22).

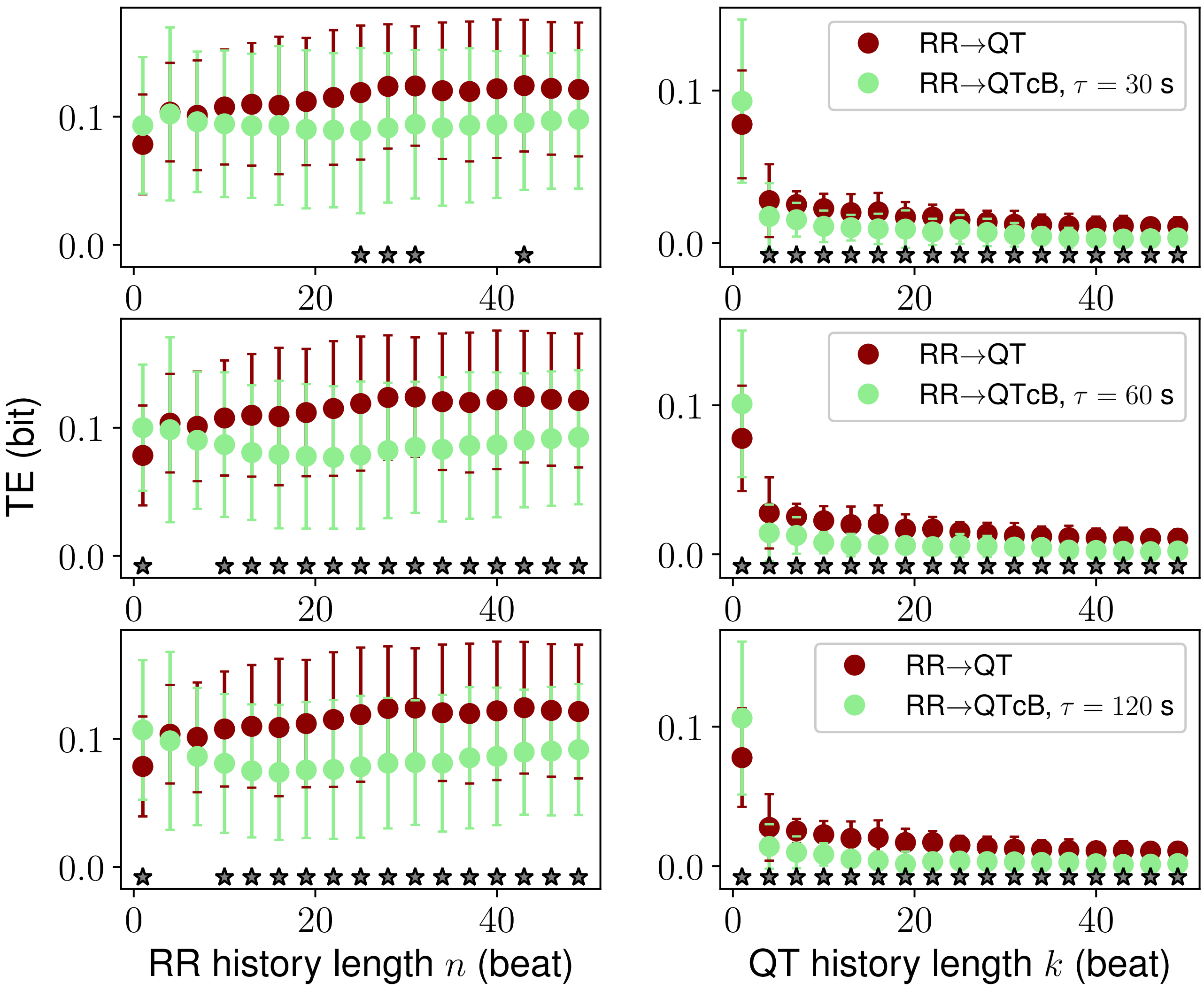

Figure S23: RR→QT and RR→QTcB (QT-corrected with the Bazett formula) transfers using the weighted average model (IR=0.183) for different time constants τ. The stars show significant ($P \leq 0.05$, paired t-test) differences between TE distributions of the original and corrected signals. Data in the format mean ± standard deviation

For IR = 0.183 (suggested in [Riad et al., 2017]) the larger reduction in $\mathrm{TE}_{\mathrm{RR}\to\mathrm{QTcB}}$ is possible. Again, for larger τ the reduction is more pronounced and reaches local minimum around $n = 20$ heartbeats of the RR history (Fig. S23).

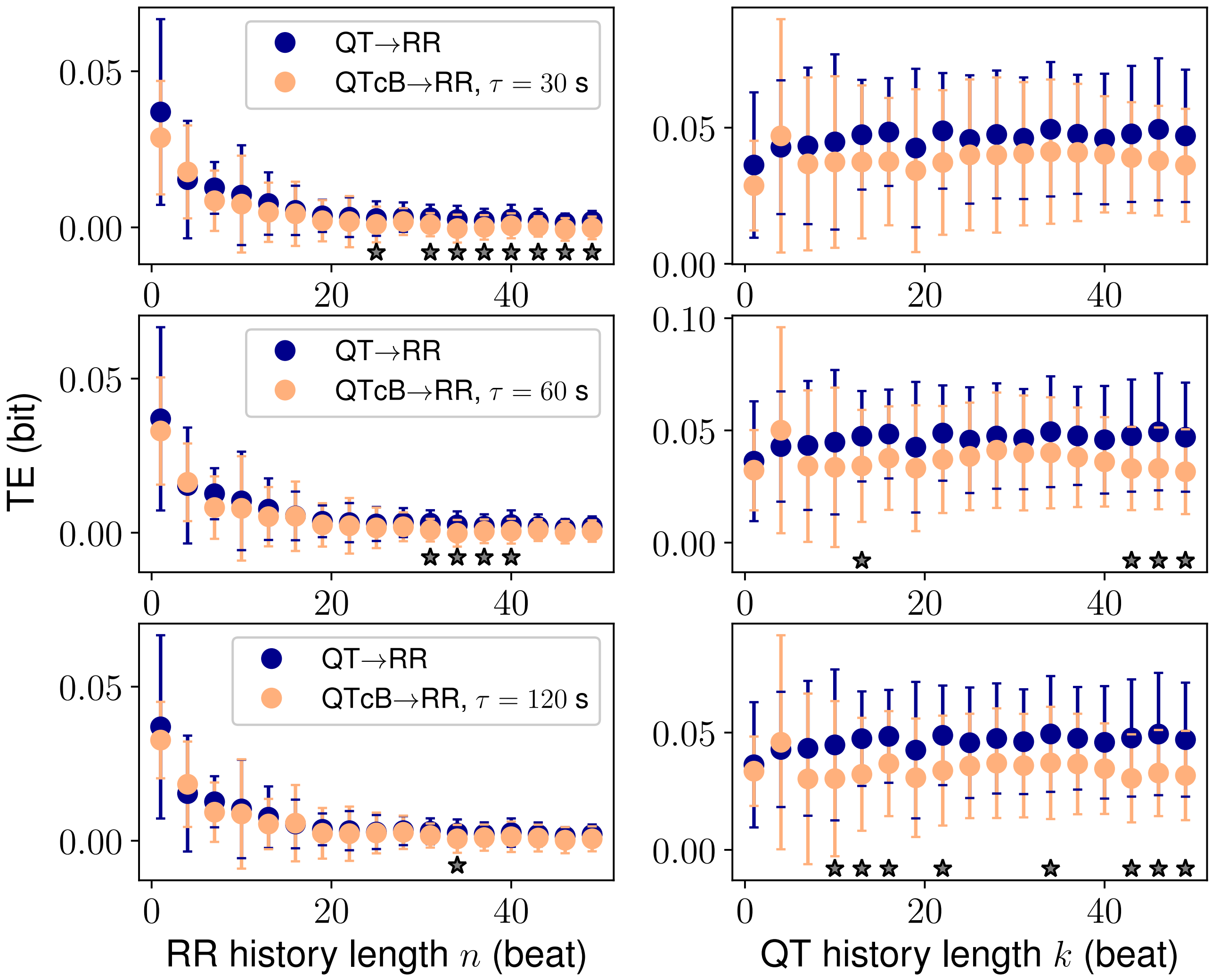

Figure S24: QT→RR and QTcB→RR (QT-corrected with the Bazett formula) transfers using the weighted average model (IR=0.183) for different time constants τ. The stars show significant ($P \leq 0.05$, paired t-test) differences between TE distributions of the original and corrected signals. Data in the format mean ± standard deviation

For IR = 0.183 $\mathrm{TE}_{\mathrm{QTcB}\to\mathrm{RR}}$ is indistinguishable from the original transfer for almost all n and k (Fig. S24), although a larger number of TE distributions demonstrates significant differences than for IR = 0.5 (cf. Fig. S22).

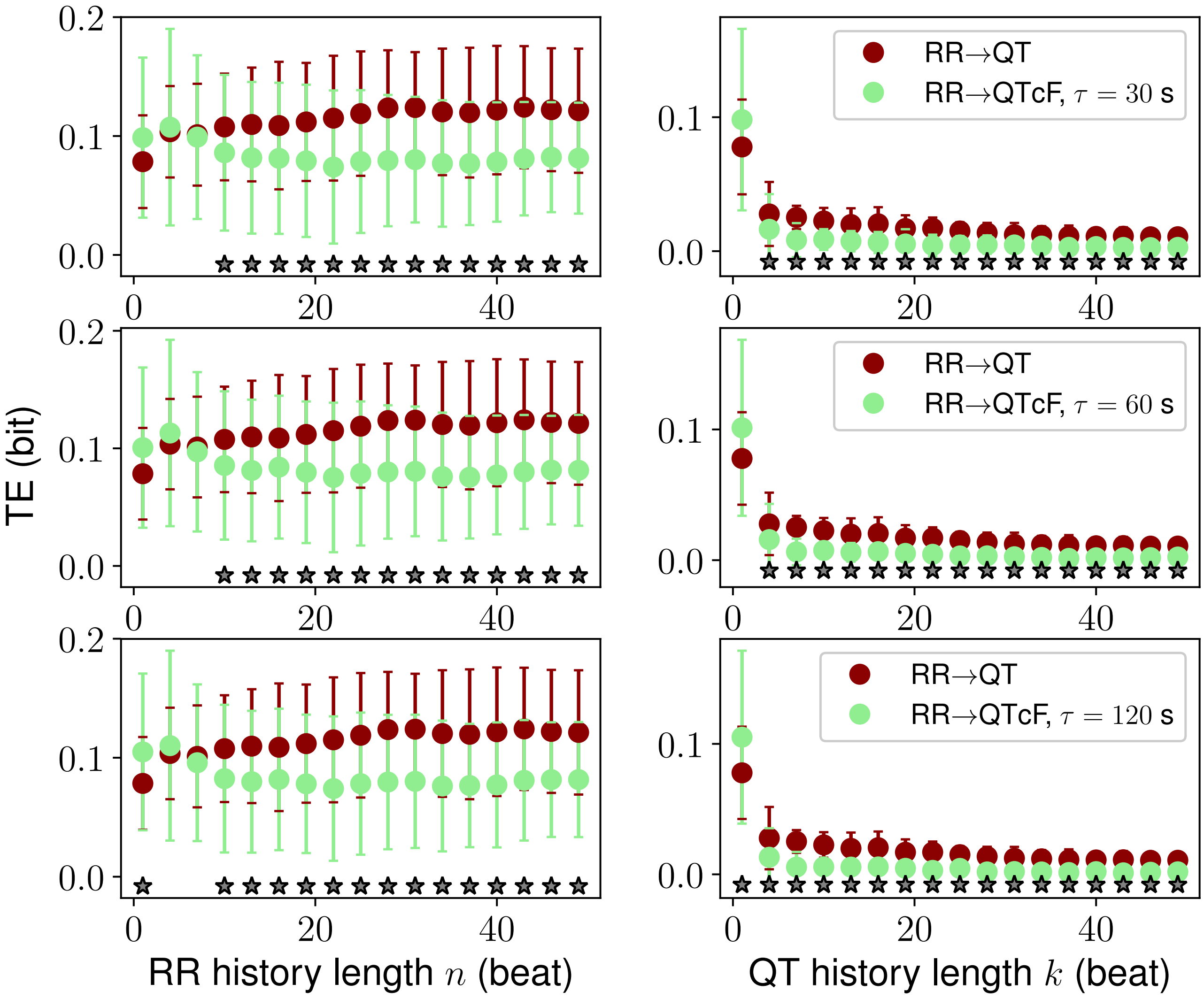

Figure S25: RR→QT and RR→QTcF (QT-corrected with the Fridericia formula) transfers using the weighted average model (IR=0.5) for different time constants τ. The stars show significant ($P \leq 0.05$, paired t-test) differences between TE distributions of the original and corrected signals. Data in the format mean ± standard deviation

The Fridericia correction shows more reduction in both RR→QTc (Figs. S25 and S27) and QTc→RR (Figs. S26 and S28) transfers than the Bazett correction for both IR = 0.5 and IR = 0.183.

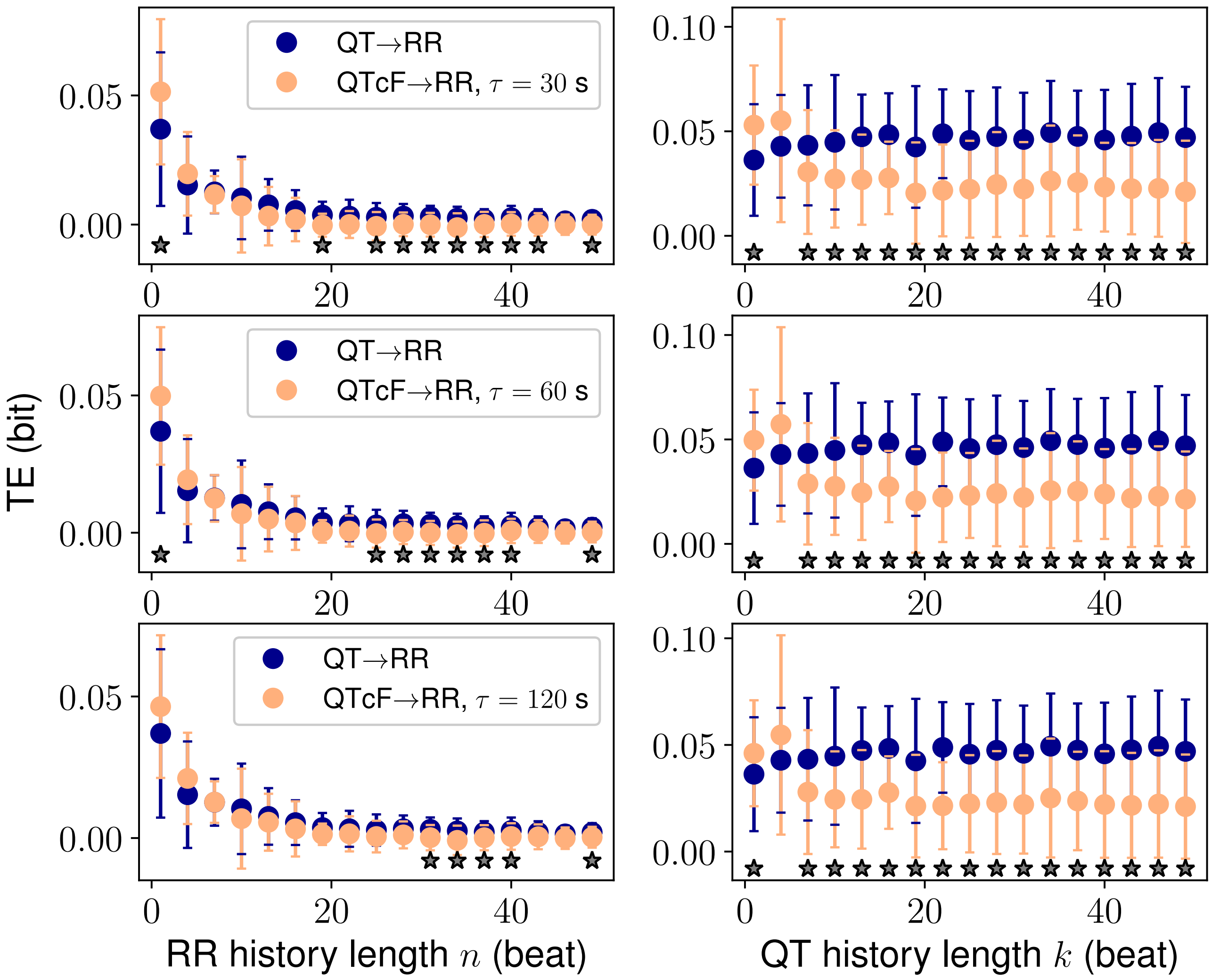

Figure S26: QT→RR and QTcF→RR (QT-corrected with the Fridericia formula) transfers using the weighted average model (IR=0.5) for different time constants τ. The stars show significant ($P \leq 0.05$, paired t-test) differences between TE distributions of the original and corrected signals. Data in the format mean ± standard deviation

For IR = 0.5 and IR = 0.183, the QTcF→RR also shows consistent reduction in the transfer levels for a larger number of QT history lengths k (Figs. S26 and S28), which was not observed for the Bazett correction (cf. Figs. S24 and S22).

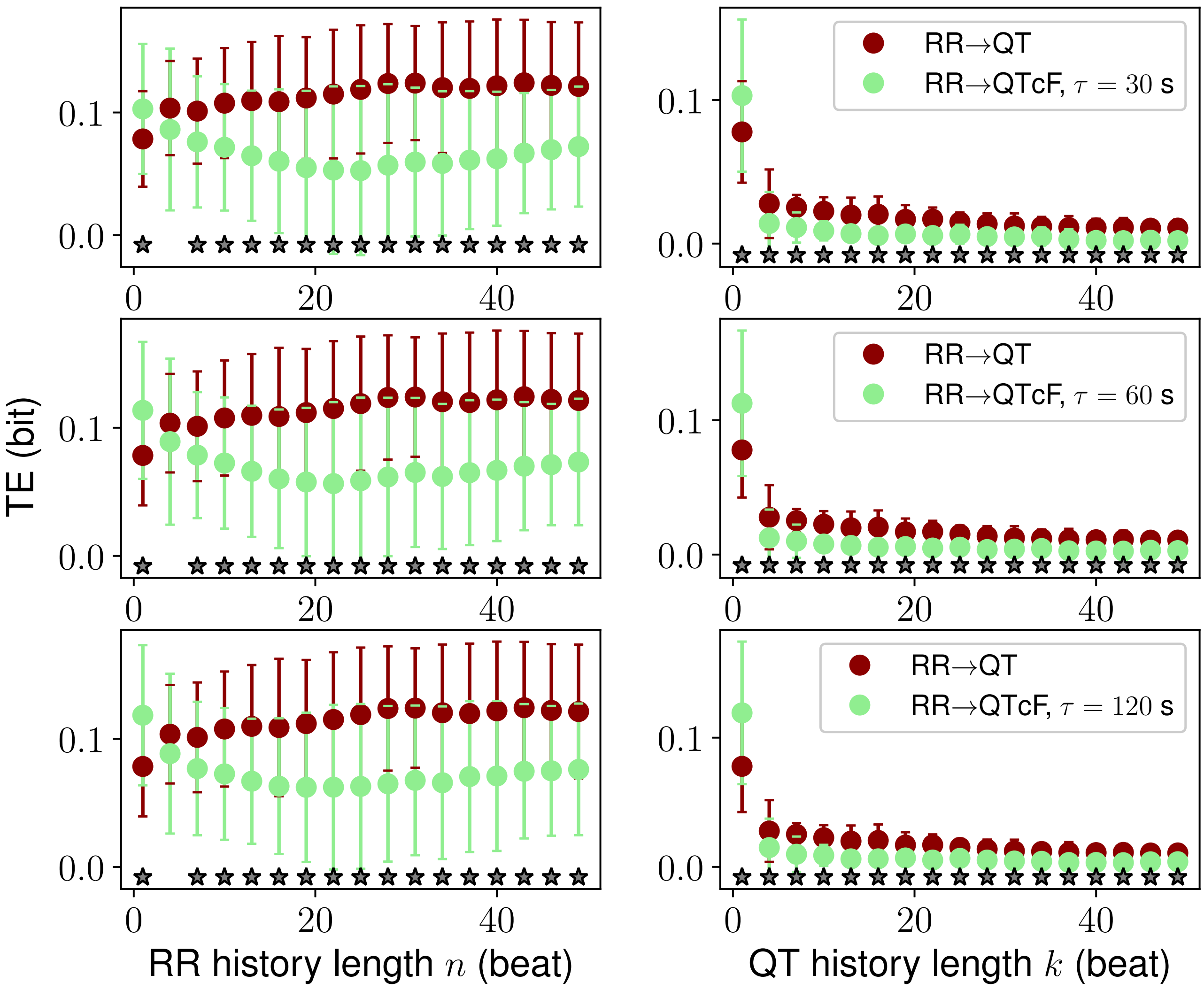

Figure S27: RR→QT and RR→QTcF (QT-corrected with the Fridericia formula) transfers using the weighted average model (IR=0.183) for different time constants τ. The stars show significant ($P \leq 0.05$, paired t-test) differences between TE distributions of the original and corrected signals. Data in the format mean ± standard deviation

The most prominent reduction in $\mathrm{TE}_{\mathrm{RR}\to\mathrm{QTc}}$ is achieved for IR $= 0.183$ and Fridericia correction (Fig. S27). However, it is not obvious if the reduction strengthens for larger τ constants, as was the case for the Bazett correction. Interestingly the minimum of the reduced $\mathrm{TE}_{\mathrm{RR}\to\mathrm{QTcF}}$ curve occurs around $n = 20$ heartbeats.

It is important to note here, that the minimum at the 20 heartbeat history length (observed here and for the Bazett correction, cf. Fig. S23) may not necessarily be connected to the physiological parameters of the heart as during the preprocessing of the data multiple consecutive beat segments were *joined* to form a single time series for an individual. Nevertheless, this characteristic length consistently appeared in several tests indicating that such a critical length might exist (see also Sec. S1.3).

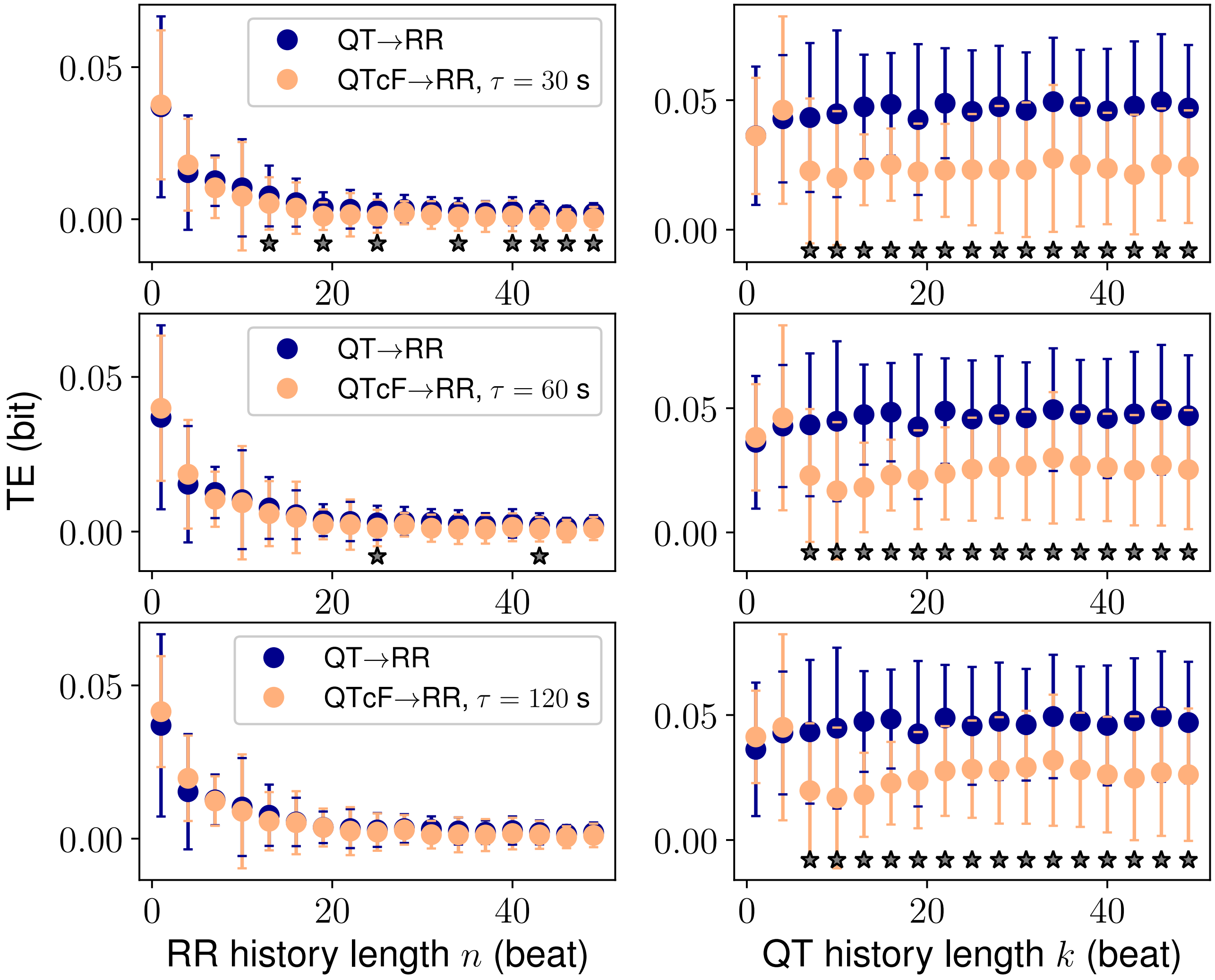

Figure S28: QT→RR and QTcF→RR (QT-corrected with the Fridericia formula) transfers using the weighted average model (IR=0.183) for different time constants τ. The stars show significant ($P \leq 0.05$, paired t-test) differences between TE distributions of the original and corrected signals. Data in the format mean ± standard deviation

S5 Information transfer and gender

We have selected male and female subgroups from the studied cohort and calculated TE distributions for different history length (Fig. S29, see the main text).

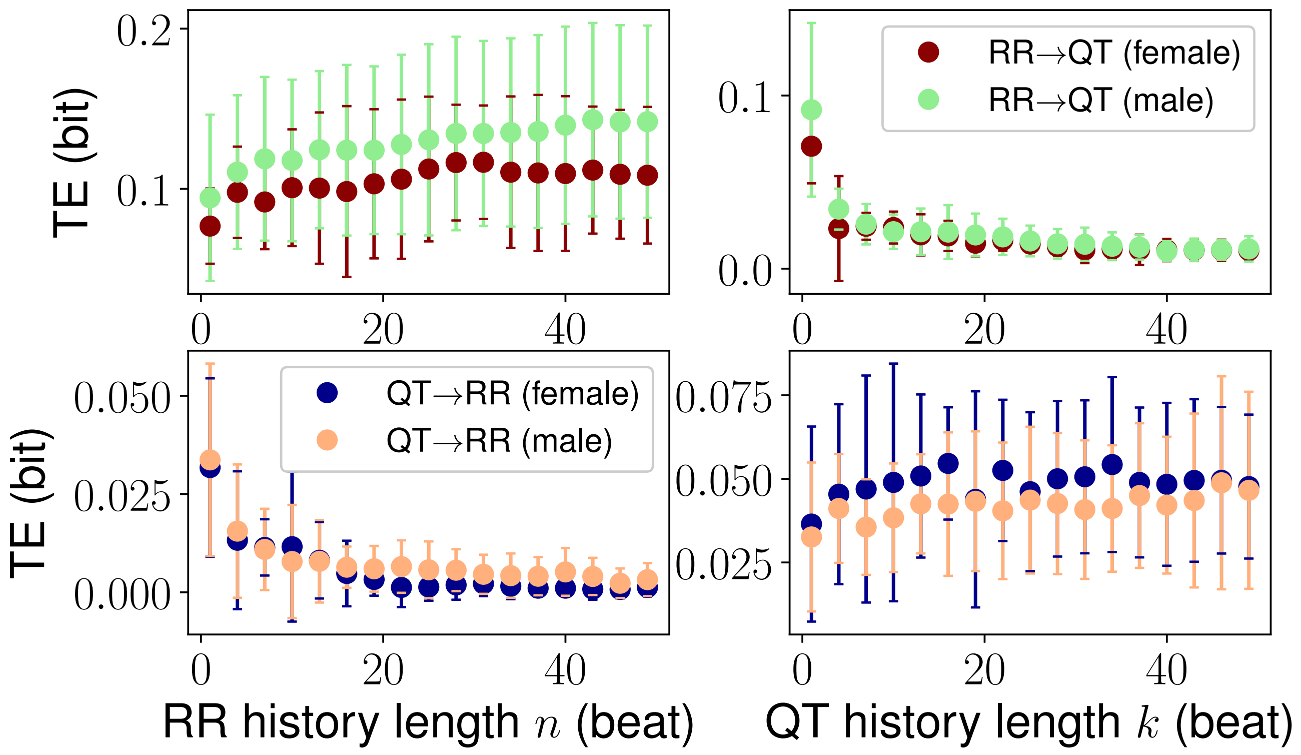

Figure S29: Gender effect on information transfer. There are no significantly different ($P \leq 0.05$, unpaired t-test) TE distributions for female and male subjects from the studied group. Data in the format mean ± standard deviation. See the main text.

S6 Information transfer in presence of a third process

We study the influence of a third process by setting a simple relation between three processes:

1. the source process (S) following the normal distribution $\mathcal{N}(800\ ms, 50\ ms)$.
2. the first recipient (R1) that receives the largest portion of influence from the source S. R1 is defined by the first order auto-regressive filter (AR1) over S.
3. the second recipient (R2) that receives a smaller portion of influence from the source S. R2 is defined by the second order auto-regressive filter (AR2) over S.

The definitions of the AR1 and AR2 processes can be found in Sec. S3.3 where the order of a process is M.

One can see that the influence of S on R1 is larger than S on R2 (the similar result was shown before in Sec. S3.3), that is, $\mathrm{TE}_{\mathrm{S}\to\mathrm{R1}} > \mathrm{TE}_{\mathrm{S}\to\mathrm{R2}}$ (Fig. S30).

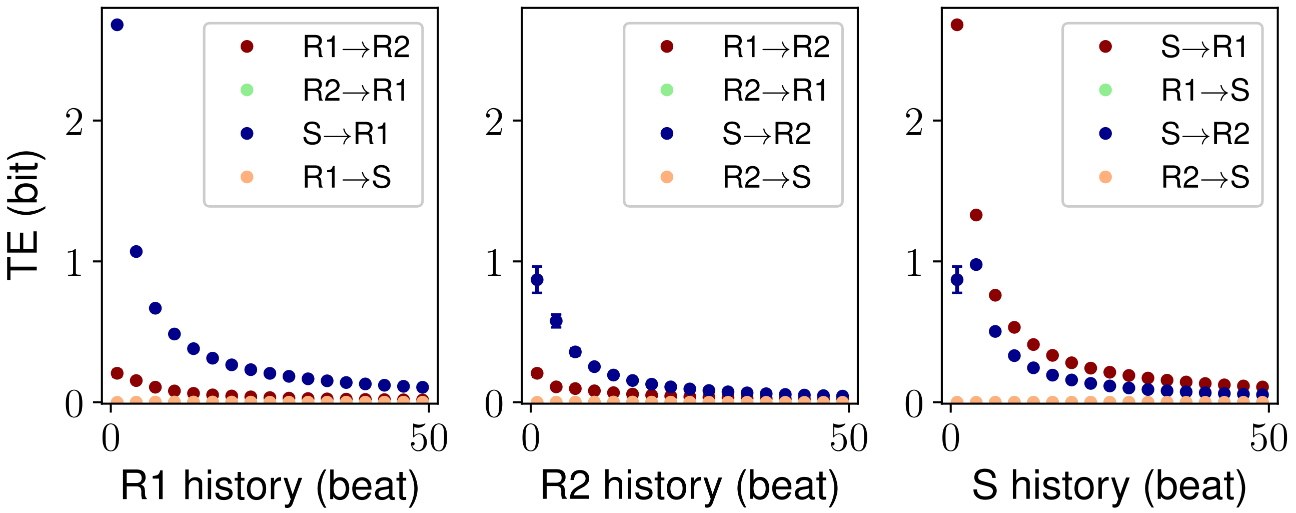

Figure S30: Information transfers between three processes: source (S), recipient 1 (R1), and recipient 2 (R2). The green curve is zero and not seen behind the orange one.

Note that $TE_{R1 \to R2} > 0$: this transfer is induced by the source S, as in absence of the source the transfer would have been zero.

The schematic representation of the information flows is shown in Fig. S31.

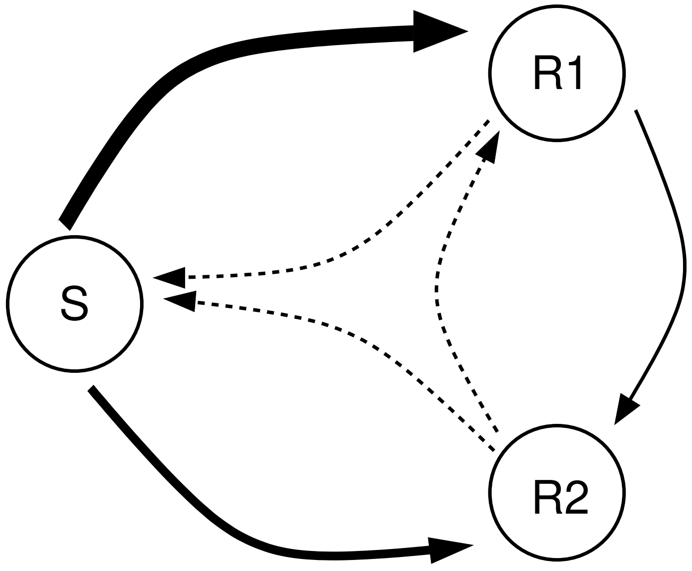

Figure S31: The schematic representation of the information flows between the source S, recipient R1, and recipient R2. The width of the arrows reflects the amount of information, dash arrows denote zero flows.

S7 List of Figures

S1 History length effect on information transfers. Dots (•) represent mean values, whereas the error bars standard deviations. Two sample size outliers with the minimal number of time samples 69 and 380 were removed, cf. Fig. 3 of the main text. Number of subjects is 16. 3

S2 History length effect when consecutive ECG segments were analyzed as independent time series. Each row shows $\mathrm{TE}_{\mathrm{RR}\to\mathrm{QT}}$ and $\mathrm{TE}_{\mathrm{QT}\to\mathrm{RR}}$ over changing RR (n) and QT (k) history lengths for a fixed parameter $\mathrm{L}_{\mathrm{min}}$, a minimal number of heartbeats in each coupled time series. The number of time series ("group") is N. 4

S3 Distribution of the group TE standard deviations after 100 runs (unpaired two-sided t-test $P = 0.029$). Each run the standard deviation of TE values over the subject group is taken to form these distributions. History length $n = k = 1$. See the complementary Fig. 2 of the main text. 5

S4 Synthetic data ($\mathrm{RR} = \mathcal{N}(800\ \mathrm{ms}, 50\ \mathrm{ms})$, $\mathrm{QT} = 300/(RR/1000)^{\frac{1}{3}}$): History length effect on information transfers. $k = 1$ and $n = 1$ on the left and right panels, respectively. Dots (•) represent mean values, whereas the error bars standard deviations. 6

S5 Same as in Fig. S4, but the number of nearest neighbors in the Kraskov-Stögbauer-Grassberger algorithm is 100. 7

S6 Synthetic data ($\mathrm{RR} = \mathcal{P}(800\ \mathrm{ms}, 50\ \mathrm{ms})$, $\mathrm{QT} = 300/(RR/1000)^{\frac{1}{3}}$): History length effect on information transfers. $k = 1$ and $n = 1$ on the left and right panels, respectively. Dots (•) represent mean values, whereas the error bars standard deviations. 8

S7 Same as in Fig. S6 but with 100 nearest neighbors of the KSG algorithm. 8

S8 Synthetic data ($\mathrm{RR} = \mathcal{N}(800\ \mathrm{ms}, 50\ \mathrm{ms})$, $\mathrm{QT_i} = \sum_{m=1}^{M} a_m \mathrm{RR}_{i-m} + \epsilon_i$, $M = 1$): History length effect on information transfers. $k = 1$ and $n = 1$ on the left and right panels, respectively. Dots (•) represent mean values, whereas the error bars standard deviations. 9

S9 Same as in Fig. S8, but $M = 2$. 10

S10 Same as in Fig. S8, but $M = 5$. 10

S11 Synthetic data ($\mathrm{RR} = \mathcal{N}(800\ \mathrm{ms}, 50\ \mathrm{ms})$, $\mathrm{QT_i} = \frac{\sum_{m=1}^{M} a_m \mathrm{RR}_{i-m} + \epsilon_i}{\sqrt{\mathrm{RR_i}/1000}}$, $M = 1$): History length effect on information transfers. $k = 1$ and $n = 1$ on the left and right panels, respectively. Dots (•) represent mean values, whereas the error bars standard deviations. 11

S12 Same as in Fig. S11, but $M = 2$. 12

S13 Same as in Fig. S11, but $M = 5$. 12

S14 A sample measured QT-RR point cloud (black) and its QT-corrected counterpart (red) with two lines showing how the Bazett formula behaves for a *fixed* QT value. 13

S15 The effect of the QT correction (Bazett formula) on the information flows. The stars show significant ($P \leq 0.05$, paired t-test) differences between TE distributions of the original and corrected signals. Data in the format mean ± standard deviation (cf. Fig. 7 of the main text). 14
S16 RR→QTcB (QT-correction with the Bazett formula) information transfers using the RR history averaging model. The stars show significant ($P \leq 0.05$, paired t-test) differences between TE distributions of the original and corrected signals. Data in the format mean ± standard deviation (cf. Fig. 7 of the main text). 15
S17 QTcB→RR information transfers using the RR history averaging model. The stars show significant ($P \leq 0.05$, paired t-test) differences between TE distributions of the original and corrected signals. Data in the format mean ± standard deviation (cf. Fig. 7 of the main text). 16
S18 RR→QTcF (QT-correction with the Fridericia formula) information transfers using the RR history averaging model. The stars show significant ($P \leq 0.05$, paired t-test) differences between TE distributions of the original and corrected signals. Data in the format mean ± standard deviation (cf. Fig. 7 of the main text). 17
S19 QTcF→RR information transfers using the RR history averaging model. The stars show significant ($P \leq 0.05$, paired t-test) differences between TE distributions of the original and corrected signals. Data in the format mean ± standard deviation (cf. Fig. 7 of the main text). 19
S20 Sample representation of the weighted average contribution of the RR history. The true history RR intervals (blue) are taken with exponentially decaying weights corresponding to three different (red, green, and black) time constants τ of the model. The straight lines represent the average RR interval to be taken for the QT-correction. 21
S21 RR→QT and RR→QTcB (QT-corrected with the Bazett formula) transfers using the weighted average model (IR=0.5) for different time constants τ. The stars show significant ($P \leq 0.05$, paired t-test) differences between TE distributions of the original and corrected signals. Data in the format mean ± standard deviation 22
S22 QT→RR and QTcB→RR (QT-corrected with the Bazett formula) transfers using the weighted average model (IR=0.5) for different time constants τ. The stars show significant ($P \leq 0.05$, paired t-test) differences between TE distributions of the original and corrected signals. Data in the format mean ± standard deviation 23
S23 RR→QT and RR→QTcB (QT-corrected with the Bazett formula) transfers using the weighted average model (IR=0.183) for different time constants τ. The stars show significant ($P \leq 0.05$, paired t-test) differences between TE distributions of the original and corrected signals. Data in the format mean ± standard deviation 24

S24 QT→RR and QTcB→RR (QT-corrected with the Bazett formula) transfers using the weighted average model (IR=0.183) for different time constants τ. The stars show significant ($P \leq 0.05$, paired t-test) differences between TE distributions of the original and corrected signals. Data in the format mean ± standard deviation . . . 25
S25 RR→QT and RR→QTcF (QT-corrected with the Fridericia formula) transfers using the weighted average model (IR=0.5) for different time constants τ. The stars show significant ($P \leq 0.05$, paired t-test) differences between TE distributions of the original and corrected signals. Data in the format mean ± standard deviation . . . 26
S26 QT→RR and QTcF→RR (QT-corrected with the Fridericia formula) transfers using the weighted average model (IR=0.5) for different time constants τ. The stars show significant ($P \leq 0.05$, paired t-test) differences between TE distributions of the original and corrected signals. Data in the format mean ± standard deviation . . . 27
S27 RR→QT and RR→QTcF (QT-corrected with the Fridericia formula) transfers using the weighted average model (IR=0.183) for different time constants τ. The stars show significant ($P \leq 0.05$, paired t-test) differences between TE distributions of the original and corrected signals. Data in the format mean ± standard deviation . . . 28
S28 QT→RR and QTcF→RR (QT-corrected with the Fridericia formula) transfers using the weighted average model (IR=0.183) for different time constants τ. The stars show significant ($P \leq 0.05$, paired t-test) differences between TE distributions of the original and corrected signals. Data in the format mean ± standard deviation . . . 29
S29 Gender effect on information transfer. There are no significantly different ($P \leq 0.05$, unpaired t-test) TE distributions for female and male subjects from the studied group. Data in the format mean ± standard deviation. See the main text. . . . 30
S30 Information transfers between three processes: source (S), recipient 1 (R1), and recipient 2 (R2). The green curve is zero and not seen behind the orange one. . . . 31
S31 The schematic representation of the information flows between the source S, recipient R1, and recipient R2. The width of the arrows reflects the amount of information, dash arrows denote zero flows. . . . 31
